# Crabp2 Promotes Metastasis of Lung Cancer Cells via HuR and Integrin β1/FAK/ERK Signaling

**DOI:** 10.1038/s41598-018-37443-4

**Published:** 2019-01-29

**Authors:** Jun-I Wu, Yi-Pei Lin, Chien-Wei Tseng, Hui-Jane Chen, Lu-Hai Wang

**Affiliations:** 1 Institute of Molecular and Genomic Medicine, National Health Research Institutes, Miaoli County, Taiwan; 2 Department of Life Sciences, National Central University, Taoyuan, Taiwan; 3 Institute of Molecular and Cellular Biology, National Taiwan University, Taipei, Taiwan; 4 Graduate Institute of Integrated Medicine, China Medical University, Taichung, Taiwan; 5 Chinese Medical Research Center, China Medical University, Taichung, Taiwan

## Abstract

Increased Crabp2 levels have been found in various types of cancer, and are associated with poor patients’ survival. Although Crabp2 is found to be overexpressed in lung cancer, its role in metastasis of lung cancer is unclear. In this study, Crabp2 was overexpressed in high-metastatic C10F4 than low-metastatic lung cancer cells. Analysis of clinical samples revealed that high CRABP2 levels were correlated with lymph node metastases, poor overall survival, and increased recurrence. Knockdown of Crabp2 decreased migration, invasion, anoikis resistance, and *in vivo* metastasis. Crabp2 was co-immunoprecipitated with HuR, and overexpression of Crabp2 increased HuR levels, which promoted integrin β1/FAK/ERK signaling. Inhibition of HuR or integrin β1/FAK/ERK signaling reversed the promoting effect of Crabp2 in migration, invasion, and anoikis resistance. Knockdown of Crabp2 further inhibited the growth of cancer cells as compared with that by gemcitabine or irinotecan alone. The expression of Crabp2 in human lung tumors was correlated with stress marker CHOP. In conclusion, our findings have identified the promoting role of Crabp2 in anoikis resistance and metastasis. CRABP2 may serve as a prognostic marker and targeting CRABP2 may be exploited as a modality to reduce metastasis.

## Introduction

Lung cancer causes more than one-fourth of all cancer-related deaths worldwide^1^. Nearly sixty percent of lung cancer patients are diagnosed at late stages with metastasis, and their 5-year survival is less than 5%^1^. Thus, identifications of novel therapeutic targets against lung cancer metastasis are urgently needed to improve patients’ survival.

Cellular retinoic acid-binding proteins, Crabp1 and Crabp2, are small cytosolic proteins that belong to a family of two isotypes^2^. CRABP1 has been found to promote tumorigenicity of transformed mesenchymal cells^3^. In breast cancer, CRABP1 is correlated with poor prognosis^4^. CRABP1 also plays a promoting role in metastasis of transformed hamster fibroblasts^3^. The overexpression of CRABP2 has been reported in tumor tissues of non-small cell lung cancer (NSCLC)^5–7^. However, the role of Crabp2 in metastasis of lung cancer is still unclear.

Metastasis is a multi-step process termed invasion-metastasis cascade, which requires multiple capabilities of cancer cells including migration and invasion^8^. Resistance to cell death induced by loss of anchorage (anoikis) has also been recognized as an essential ability for metastasis^9,10^. Further studies revealed that anoikis resistance is closely related to migration and invasion. Selection of anoikis-resistant pancreatic cancer cells results in enhanced cell migration and invasion^11^. Elevated migration and invasion were also found in anoikis-resistant prostate cancer cells^12^. It has been reported that activation of integrin signaling molecules including FAK and ERK is known to promote anoikis resistance, migration, invasion, and metastasis of cancer cells^13–16^, and both FAK and ERK are thus suggested as therapeutic targets^17,18^ while side effects disturbing normal cell functions have also been reported^19^. Thus, identification of tumor-overexpressing molecules mediating the activation of integrin signaling and promotion of lung cancer metastasis is needed.

In this study, we selected the high-metastatic C10F4 lung cancer cells from low-metastatic C9F6 lung adenocarcinoma cells. Further analyses identified Crabp2 as an overexpressed gene in C10F4 cells in comparison with C9F6 cells and mouse lung cells. Multiple cohorts of lung cancer patients were analyzed to reveal the correlation of CRABP2 with tumor progression and clinical outcomes. We further explored the role of Crabp2 in migration, invasion, anoikis resistance, and *in vivo* metastasis. The signaling regulated by Crabp2 was investigated, and their roles in Crabp2-mediated pro-metastatic features were examined. We then addressed the potential implication of Crabp2 knockdown in inhibiting the growth of cancer cells as compared with that by gemcitabine or irinotecan alone. We also explored the potential upstream regulating factors leading to the upregulation of Crabp2 in lung cancer cells. Overall, our findings reveal the promoting role of Crabp2 in migration, invasion, anoikis resistance, and metastasis of lung cancer. CRABP2 could be a useful prognostic biomarker and a target against lung cancer metastasis.

## Results

### Establishment of high-metastatic C10F4 lung cancer cells

**Figure 1:**
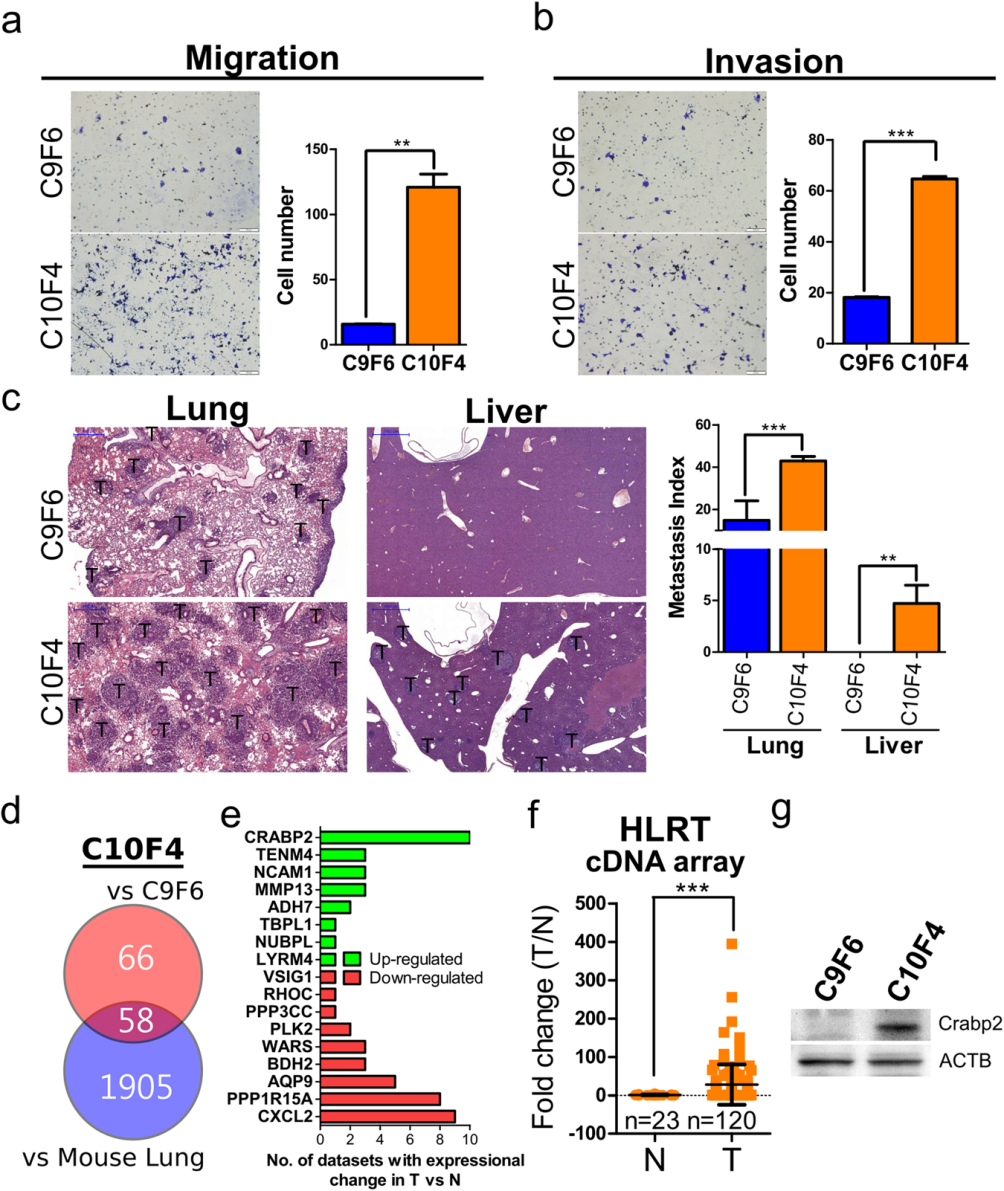
Crabp2 is overexpressed in high-metastatic C10F4 cells. (**a**) Migration assay of C9F6 and C10F4 cells for 12 hours. Cells migrated into the lower compartment of Boyden chamber were photographed (left) and quantified (right). (**b**) Matrigel cell invasion assay of C9F6 and C10F4 for 15 hours. Cells invaded through the matrigel were photographed (left) and quantified (right). (**c**) Metastasis of C9F6 (n = 3) and C10F4 (n = 3) cells. One million cells were injected into tail veins of each BALB/c mouse. Twelve days later, mouse lungs and livers were harvested, and tumor regions were visualized by H&E staining (left). Metastasis index was calculated as tumor area over lung/liver area (right). T: tumor region. (**d,e**) Venn diagram comparing two differential expression analyses. For the up red circle, 124 genes were expressed more than 2-fold-higher in C10F4 cells than C9F6 cells from exon array results. For the down blue circle, 1963 genes were expressed more than 2-fold higher in C10F4 cells than normal lung cells from mouse in exon array results. The intersection identified 58 C10F4-overexpressing genes compared to both C9F6 and normal lung cells (**d**). Seventeen out of 58 genes exhibited significant differential expression in lung tumors when compared with normal tissues from Oncomine datasets, including 8 up-regulated (green) and 9 down-regulated (red) genes (**e**). (**f**) CRABP2 levels in tumors (n = 120) versus normal lungs (n = 23) of lung cancer patients were detected by real-time PCR (*p* < 0.001). Samples were from HLRT Tissuescan cDNA arrays, and the statistical significance was determined using Mann-Whitney test. (**g**) Western blot analysis of Crabp2 and β-actin (ACTB, as the loading control) in C9F6 and C10F4 cells. **p < 0.01, ***p < 0.001.

We initially used *in vivo* tail vein injection selection to obtain a high-metastatic subline. Three cycles of tail vein injection selection yielded the highly metastatic C10F4 cells from low-metastatic C9F6 cells. We further compared metastatic behaviors, including migration and invasion, in C10F4 and C9F6 cells. The C10F4 cells displayed significantly enhanced migration and invasion ability compared to C9F6 cells (Fig. 1a,b). The BALB/c mice tail vein injection model showed that C10F4 cells exhibited higher lung and liver metastatic abilities than C9F6 cells (Fig. 1c). Thus C10F4 line provides us with a valuable tool for exploring metastasis-related signaling pathways and molecules.

### Crabp2 is overexpressed in high-metastatic C10F4 lung cancer cells

To identify genes that are potentially responsible for the enhanced metastasis of C10F4 cells but are not overexpressed in normal lung cells, we compared C10F4 cells with C9F6 cells and with normal lung cells from mouse using exon array. 124 genes were identified to be expressed higher in C10F4 cells than in C9F6 cells and 1963 genes were expressed higher in C10F4 than normal cells (Fig. 1d). 58 genes were expressed higher in C10F4 cells when compared with C9F6 and normal cells (Fig. 1d). Among those 58 genes, further analysis identified 17 genes with significant differential expression in tumor tissues versus normal ones using Oncomine datasets^20^, including eight up- and nine down-regulated genes (Fig. 1e). Clinical analysis from ten Oncomine datasets showed that CRABP2 was overexpressed in lung tumors than normal lungs (Fig. 1e), and we also observed similar results using commercial human tissue cDNA array (Fig. 1f, Table S1). Western blot analysis revealed that Crabp2 was overexpressed in C10F4 than C9F6 cells (Fig. 1g). These results suggest that Crabp2, a tumor-overexpressing gene, is overexpressed in high-metastatic C10F4 lung cancer cells.

### CRABP2 is associated with tumor progression, poor overall survival, and recurrence of lung cancer patients

**Figure 2:**
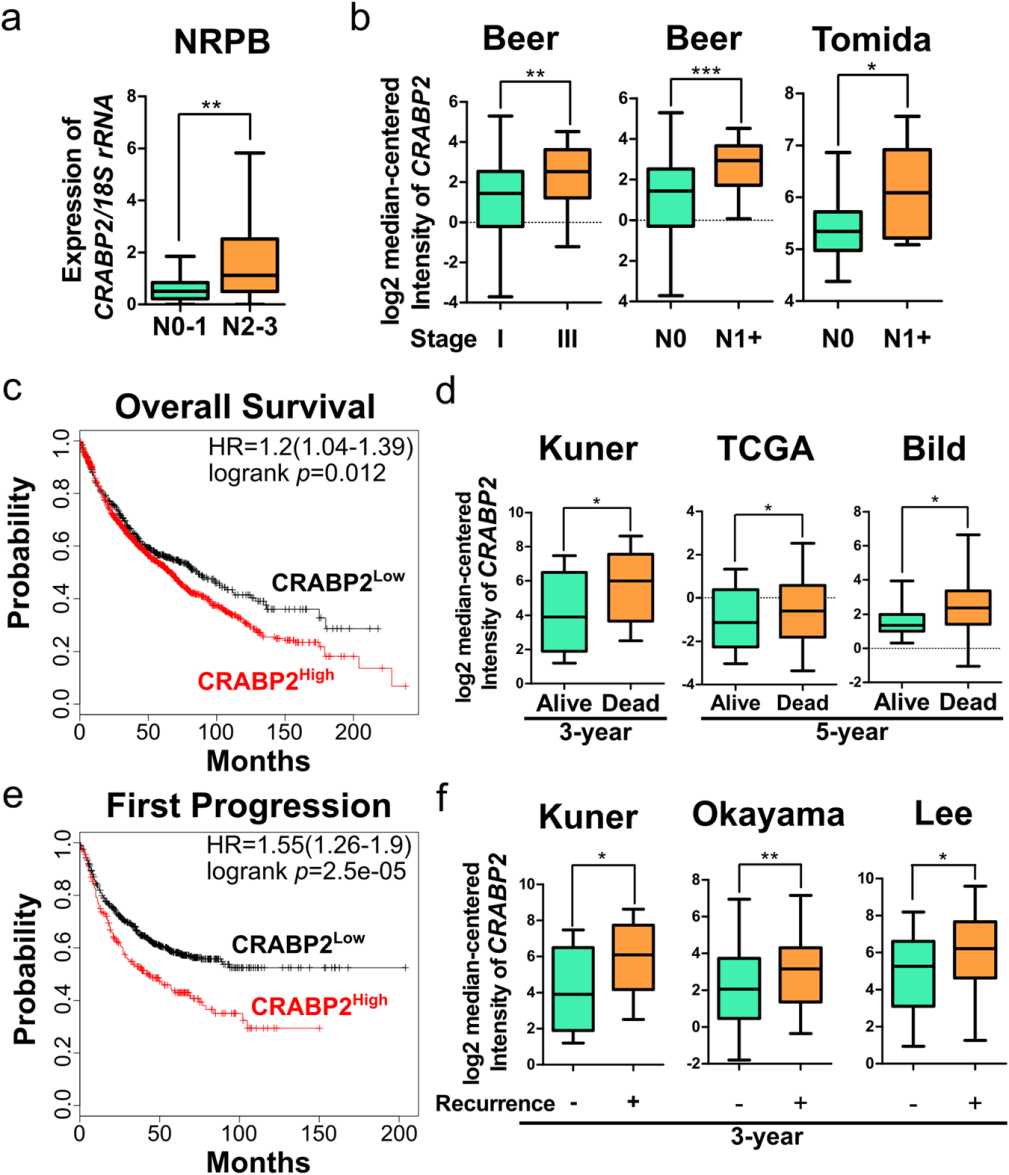
CRABP2 is associated with tumor progression, poor survival, and recurrence of lung cancer patients. (**a**) CRABP2 levels in blood buffy coat RNA samples of N0-N1 patients (n = 12) versus N2-N3 patients (n = 36) were detected by real-time RT-PCR (*p* = 0.0089). The *p* value was determined using Mann-Whitney test. (**b**) Levels of CRABP2 in stage I versus stage III lung tumors, or N0 versus N1 + lung tumors using data from Beer Lung (lung adenocarcinoma, n = 86) or Tomida Lung (lung adenocarcinoma, n = 30). The *p* values are from the Oncomine database. (**c**,**e**) Kaplan-Meier plot of the overall survival of 1926 lung cancer patients (**c**), or the first progression after surgery of 982 lung cancer patients (**e**) stratified by CRABP2 expression level using data from Kaplan-Meier Plotter database. Patients with high CRABP2 levels (CRABP2^High^) exhibited higher risks than patients with low CRABP2 levels (CRABP2^Low^) in both overall survival (HR = 1.2, logrank *p* = 0.012) and first progression after surgery (HR = 1.55, logrank *p* = 2.5e-5) (optimal cutoff was provided by Kaplan-Meier Plotter database). (**d**) Levels of CRABP2 in lung tumors of patients alive or dead at 3 years (Kuner) or 5 years (TCGA and Bild) after diagnosis using data from Kuner Lung (lung adenocarcinoma, n = 31), TCGA (squamous cell lung carcinoma, n = 73), and Bild Lung (squamous cell lung carcinoma, n = 35), and the *p* values are from the Oncomine database. (**f**) Levels of CRABP2 in lung tumors of patients showing recurrence or not at 3 years after diagnosis using data from Kuner Lung (lung adenocarcinoma, n = 30), Okayama Lung (lung adenocarcinoma, n = 207), and Lee Lung (lung adenocarcinoma, n = 56), and the *p* values are from the Oncomine database. *p < 0.05, **p < 0.01, ***p < 0.001.

We next explored the correlation of CRABP2 levels with tumor progression of lung cancer patients. Using blood buffy coat samples from lung cancer patients (n = 48), our results showed significantly higher CRABP2 levels in patients with advanced lymph node metastasis (N2-3) than those having none or early lymph node metastasis (N0-1) (*p* = 0.0089, Fig. 2a). Similarly, higher CRABP2 levels were found in tumor tissues of stage III than in stage I patients, and also higher in lymph node metastatic (N1+) than non-metastatic (N0) patients (Fig. 2b). These results suggest the potential involvement of CRABP2 in tumor progression.

Metastasis is the major cause of cancer-related death^21^, and metastatic tumors are the source of recurrence in lung cancer patients including those had surgical resection^22^. As Crabp2 is overexpressed in high-metastatic C10F4 cells, and is correlated with tumor progression of lung cancer patients, we further evaluated the association of CRABP2 with prognosis and recurrence of lung cancer patients. Kaplan Meier survival curves revealed that high CRABP2 levels in lung tumors were significantly associated with poor overall survival (*p* = 0.012, Fig. 2c)^23^. Similarly, multivariate survival analysis of CRABP2 and other known lung cancer prognostic markers, including ribonucleotide reductase messenger 1 (RRM1), excision repair cross-complementation group (ERCC1) and p27^24,25^, also indicated that high CRABP2 levels in lung tumors were significantly associated with poor overall survival (*p* = 0.0306, Tables S2–5). Higher CRABP2 levels were also found to be correlated with decreased patients’ survival in three cohorts of lung cancer tissues (Fig. 2d). Furthermore, high CRABP2 levels in lung tumors were significantly associated with the first progression after surgery (*p* = 2.5e-5, Fig. 2e)^23^. A significant correlation of high CRABP2 levels with the first progression after surgery was also found using multivariate analysis (*p* = 0.0074, Table S2). Higher CRABP2 levels were found to be associated with recurrence in three cohorts of tumor tissues (Fig. 2f). These results suggested the correlation of CRABP2 with poor overall survival and recurrence of lung cancer patients.

### Knockdown of Crabp2/CRABP2 suppresses migration, invasion, anoikis resistance, and *in vivo* metastasis

**Figure 3:**
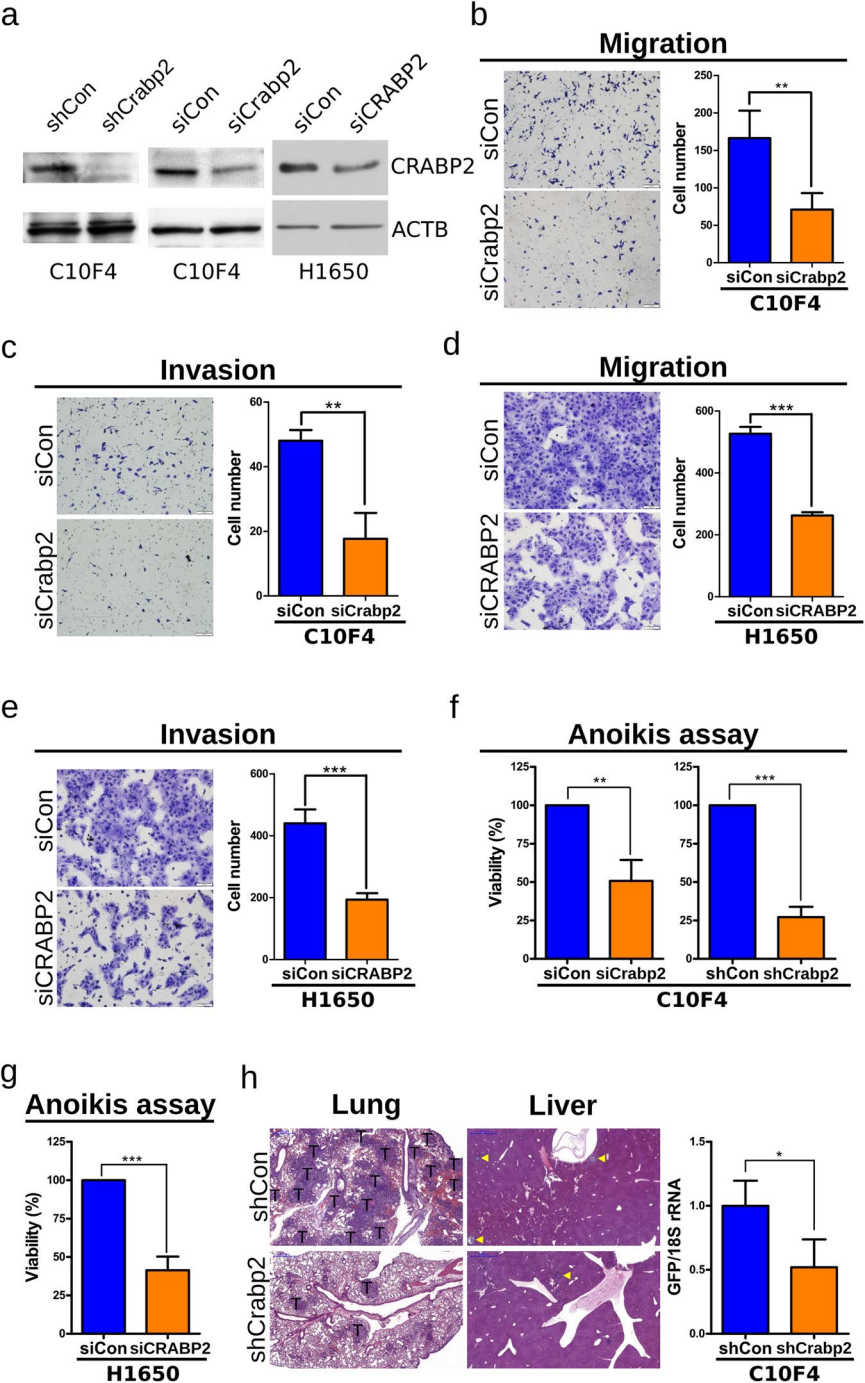
Knockdown of Crabp2/CRABP2 suppresses migration, invasion, anoikis resistance, and *in vivo* metastasis. (**a**) Western blot analysis of Crabp2/CRABP2 and β-actin (ACTB, as the loading control) in C10F4 or H1650 cells expressing control-shRNA (shCon) versus Crabp2-shRNA (shCrabp2) (left), control-siRNA (siCon) versus Crabp2/CRABP2-siRNA (siCrabp2/siCRABP2) (middle and right). (**b**,**d**) Migration assay of C10F4 (**b**) or H1650 (**d**) cells expressing control-siRNA versus Crabp2/CRABP2-siRNA for 12 hours. Cells migrated into the lower compartment of Boyden chamber were photographed (left) and quantified (right). (**c**,**e**) Matrigel cell invasion assay of C10F4 (**c**) or H1650 (**e**) cells expressing control-siRNA versus Crabp2/CRABP2-siRNA for 15 (C10F4) or 12 (H1650) hours. Cells invaded through the matrigel were photographed (left) and quantified (right). (**f**) Anoikis assay of C10F4 cells expressing control-siRNA versus Crabp2-siRNA (left), or control-shRNA versus Crabp2-shRNA (right). Cells were plated onto a 96-well anchorage-resistant plate at a density of 10,000 cells/well. Twenty-four hours later, viability of cells was assessed by MTS assay. The *p* values were determined using Student’s t test. (**g**) Anoikis assay of H1650 cells expressing control-siRNA versus CRABP2-siRNA. Cells were plated onto a 96-well anchorage-resistant plate at a density of 10,000 cells/well. Twenty-four hours later, viability of cells was assessed by MTS assay. The *p* value was determined using Student’s t test. (**h**) Metastasis of GFP^+^ C10F4 cells expressing control-shRNA (n = 3) versus Crabp2-shRNA (n = 3). One million cells were injected into tail veins of each BALB/c mouse. Twelve days later, mouse lungs and livers were harvested and analyzed using H&E staining (left), and metastatic cells at lung were analyzed using real-time RT-PCR of GFP RNAs (right). Tumor regions were indicated by T or yellow triangle. The *p* value was determined using Student’s t test. *p < 0.05, **p < 0.01, ***p < 0.001.

Reports showed that CRABP2 promoted proliferation of glioblastoma and malignant peripheral nerve sheath tumor cells^26,27^. We found that knockdown of Crabp2 by lentiviral shRNA (shCrabp2, Fig. 3a) inhibited the proliferation of C10F4 cells (Fig. S1). At present, the role of Crabp2 in metastasis of lung cancer has not been investigated. We then explore the role of Crabp2 in metastasis and found knockdown of Crabp2 (Fig. 3a) inhibited the migration and invasion of C10F4 cells (Fig. 3b,c). Similarly, siRNA of CRABP2 reduced the migration and invasion of metastatic human lung adenocarcinoma H1650 cells (Fig. 3d,e). These results suggest that Crabp2 plays an important role for migration and invasion of C10F4 and H1650 cells. Resistance to anoikis has been recognized as an essential ability to facilitate tumor dissemination^9,10^. Using the anoikis resistance assay, knockdown of Crabp2 by short-hairpin RNA or siRNA reduced the viability of non-adherent C10F4 cells (Fig. 3f). Similarly, knockdown of CRABP2 reduced the anoikis resistance of H1650 cells (*p* < 0.001, Fig. 3g). Thus, knockdown of Crabp2/CRABP2 inhibited the anoikis resistance, migration, and invasion of C10F4 and H1650 cells.

We next used tail vein injection model of BALB/c mice to evaluate the role of Crabp2 in metastasis *in vivo*. C10F4 cells expressing control-shRNA (shCon) or Crabp2-shRNA (shCrabp2) were labeled with GFP by lentiviral infection and were injected into tail veins of syngeneic BALB/c mice, respectively. Twelve days later, mouse lungs and livers were harvested and the presence of tumor cells was validated by H&E staining or real-time RT-PCR of GFP RNA. Knockdown of Crabp2 significantly reduced the metastatic ability of C10F4 cells (*p* = 0.0234, Fig. 3h). Together, our results for the first time identified the metastasis-promoting role of Crabp2 in lung cancer.

### Crabp2 promotes integrin β1/FAK/ERK signaling via HuR

The mechanism of how Crabp2 promotes metastatic capability is still unclear. We next explored the mechanism that potentially contributing to the Crabp2-mediated effects on metastatic abilities. Crabp2 is known to deliver retinoic acid (RA) to the nucleus, where RA binds to transcription factors to regulate transcription of downstream genes^28^. We tested if RA can affect anoikis resistance via Crabp2 and found the inhibitory effect of retinoic acid on anoikis resistance is irrelative to Crabp2 in C10F4 cells (Fig. S2). Thus, it appears that retinoic acid is not involved in the regulation of anoikis resistance by Crabp2.

**Figure 4:**
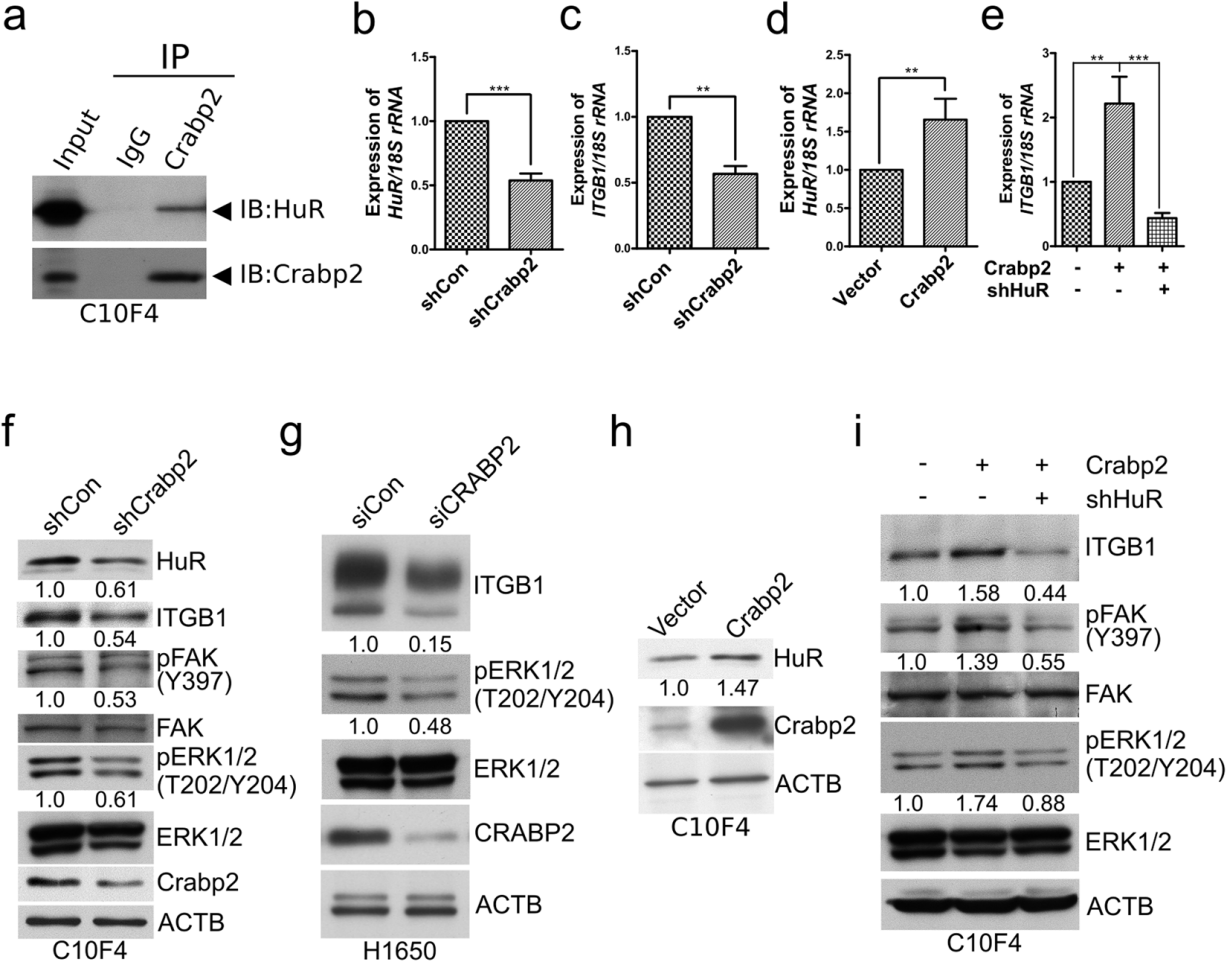
Crabp2 promotes integrin β1/FAK/ERK signaling via HuR. (**a**) C10F4 cell lysate was immunoprecipitated with Crabp2 antibody or rabbit IgG (as control), respectively. Western blot analysis was performed to detect Crabp2 and HuR in input lysate (Input) and co-immunoprecipitated proteins. (**b** and **d**) Real-time RT-PCR of HuR in C10F4 cells expressing control-shRNA (shCon) versus Crabp2-shRNA (shCrabp2) (**b**), or C10F4 cells expressing empty vector versus Crabp2 (**d**). The *p* values were determined using Student’s t test. (**c**,**e**) Real-time RT-PCR of integrin β1 (Itgb1) in C10F4 cells expressing control-shRNA or Crabp2-shRNA (**c**), or C10F4 cells expressing Vector + shCon, Crabp2 + shCon, or Crabp2 + shHuR (**e**). The *p* values were determined using Student’s t test (**c**) or one-way ANOVA (**e**). **p < 0.01, ***p < 0.001. (**f**) Western blot analysis of HuR, integrin β1 (ITGB1), phosphorylated FAK (Y397), total FAK, phosphorylated ERK1/2 (T202/Y204), total ERK1/2, Crabp2, and β-actin (ACTB, as the internal control) levels in C10F4 cells expressing control-shRNA or Crabp2-shRNA. (**g**) Western blot analysis of integrin β1, phosphorylated ERK1/2 (T202/Y204), total ERK1/2, CRABP2, and β-actin (ACTB, as the internal control) levels in H1650 cells expressing control-siRNA or CRABP2-siRNA. (**h**) Western blot analysis of HuR, Crabp2, and β-actin (ACTB, as the internal control) in C10F4 cells expressing empty vector or Crabp2. (**i**) Western blot analysis of integrin β1 (ITGB1), phosphorylated FAK (Y397), total FAK, phosphorylated ERK1/2 (T202/Y204), total ERK1/2, and β-actin (ACTB, as the internal control) levels in C10F4 cells expressing Vector + shCon, Crabp2 + shCon, or Crabp2 + shHuR.

Previous studies have reported that Crabp2 regulates HuR (ELAV-like protein 1) expression in breast cancer^29,30^. In addition, the association between Crabp2 and HuR has been shown by co-immunoprecipitation^30^. HuR has been proposed to promote invasion and metastasis^31^ and is known to correlates with lymph node metastasis of non-small cell lung cancer patients^32^. Moreover, knockdown of HuR inhibited migration and invasion of lung cancer cells^33^ and anoikis resistance of immortalized breast epithelial cells^34^. Our results showed that HuR co-immunoprecipitated with Crabp2 in C10F4 cells (Fig. 4a). Knockdown of Crabp2 inhibited HuR and integrin β1 expression (Fig. 4b,c), a known downstream molecule of HuR in Jurkat T cells^35^ and was reported to promote lung cancer migration, invasion, and metastasis^36^. The opposite effect was observed in C10F4 cells transfected with the Crabp2-overexpressing plasmid, which led to enhanced HuR and integrin β1 levels (Fig. 4d,e). Moreover, HuR knockdown reversed the effects of Crabp2 overexpression on integrin β1 expression (Figs 4e, S3). These results suggest that HuR is needed by Crabp2 to promote integrin β1 expression.

Integrin signaling is known to promote anoikis resistance, migration, and invasion^37^. We next explored the role of Crabp2 in the regulation of integrin signaling. FAK is one of the most important integrin signaling molecules. Activation of FAK, as indicated by its phosphorylation at Y397, is known to be correlated with tumor progression^38^, and is known to be critical to achieve resistance to anoikis and cancer invasiveness^14,39^. Similarly, activation of ERK, another important downstream mediator in integrin signaling, is known to promote cancer migration, invasion, and anoikis resistance^16,40^. Knockdown of Crabp2 inhibited the phosphorylation of FAK-Y397 and ERK (T202/Y204) (Fig. 4f,g). Conversely, overexpression of Crabp2 increased phosphorylation of FAK-Y397 and ERK (T202/Y204), which could be reversed by HuR knockdown (Fig. 4h,i). These results suggest that Crabp2 promotes integrin β1/FAK/ERK signaling via HuR.

### Crabp2 promotes migration, invasion, and anoikis resistance via HuR and integrin β1/FAK/ERK signaling

**Figure 5:**
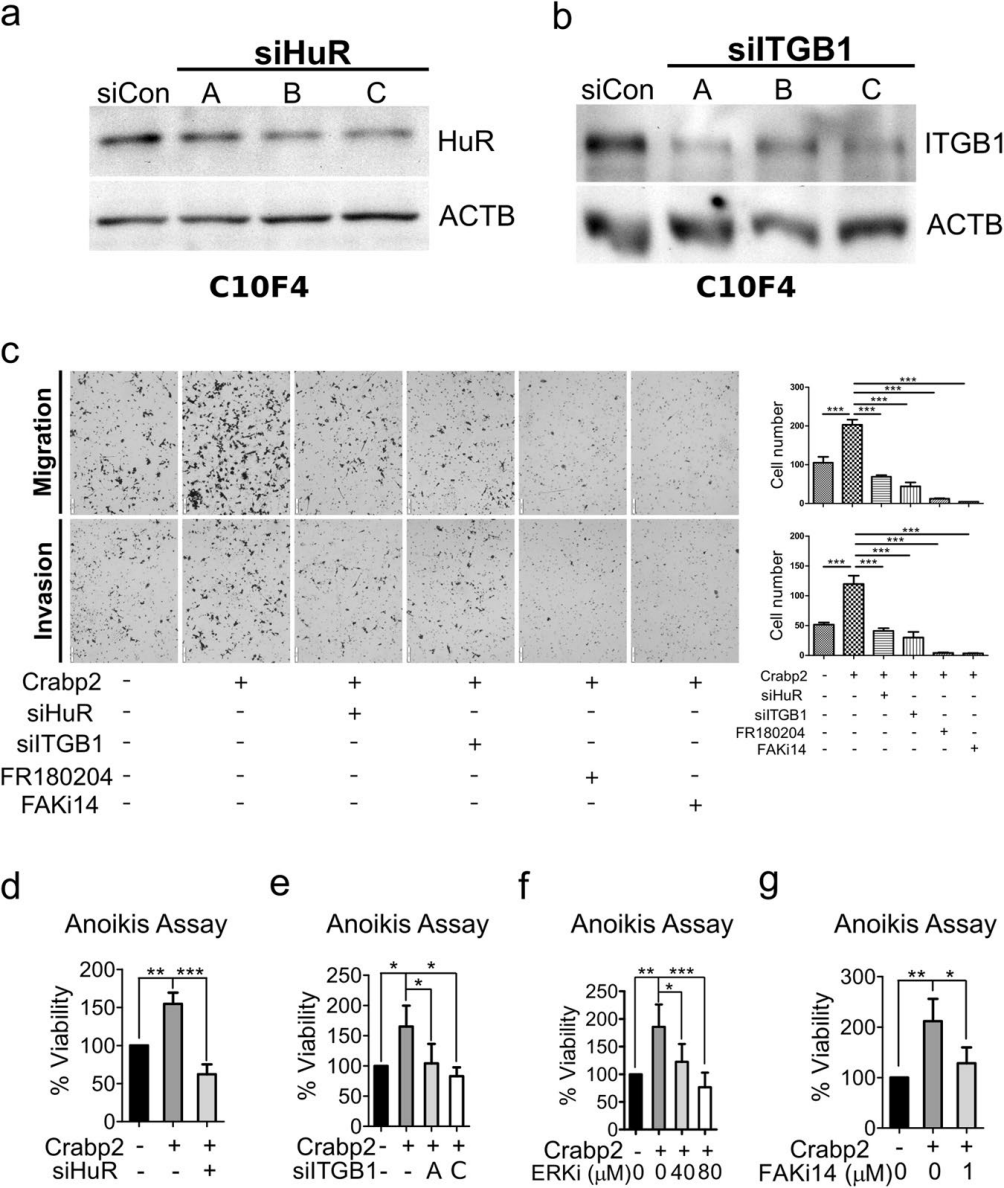
Crabp2 promotes migration, invasion, and anoikis resistance via HuR and integrin β1/FAK/ERK signaling. (**a**) Western blot analysis of HuR of C10F4 cells expressing control-siRNA or HuR-siRNA (A, B, or C). (**b**) Western blot analysis of integrin β1 (ITGB1) of C10F4 cells expressing control-siRNA or ITGB1-siRNA (A, B, or C). (**c**) Migration (up) or invasion (down) assay of C10F4 cells expressing Vector + siCon, Crabp2 + siCon, Crabp2 + siHuR (siHuR-C), Crabp2 + siITGB1 (siITGB1-A), Crabp2 + FR180204 (40 µM), or Crabp2 + FAK inhibitor 14 (1 µM). Cells migrated into the lower compartment of Boyden chamber were photographed (left) and quantified (right). The *p* values were determined using one-way ANOVA. (**d,e**) Anoikis assay or C10F4 cells expressing Vector + siCon, Crabp2 + siCon, or Crabp2 + siHuR (siHuR-C) (**d**), or C10F4 cells expressing Vector + siCon, Crabp2 + siCon, or Crabp2 + siITGB1-A/C (**e**). Cells were plated onto a 96-well anchorage-resistant plate at a density of 10,000 cells/well. Twenty-four hours later, viability of cells was assessed by MTS assay. The *p* values were determined using one-way ANOVA. (**f,g**) C10F4 cells expressing empty vector or Crabp2 were plated onto a 96-well anchorage-resistant plate at a density of 10,000 cells/well and treated with DMSO (as control), ERK1/2 inhibitor FR180204 (ERKi, 40 or 80 μM) (**f**), or FAK inhibitor 14 (FAKi14, 1 μM) (**g**). Twenty-four hours later, viability of cells was assessed by MTS assay. The *p* values were determined using one-way ANOVA *p < 0.05, **p < 0.01, ***p < 0.001.

We next aimed to address the involvement of HuR as well as integrin β1/FAK/ERK signaling in Crabp2-mediated promoting effect on migration, invasion, and anoikis resistance. Here we transfected Crabp2-overexpressing plasmid in C10F4 cells, followed by subsequent knockdown of HuR or integrin β1 (Fig. 5a,b), or treatment with inhibitor of ERK (FR180204) or FAK-Y397 (FAK inhibitor 14). The results showed that overexpression of Crabp2 promoted the migration, invasion, and anoikis resistance of C10F4 cells (Fig. 5c,d). Knockdown of HuR, or inhibition of integrin signaling by integrin β1-siRNA or FAK/ERK inhibitors reversed the promoting effect of Crabp2 in migration, invasion, and anoikis resistance (Fig. 5c–g). Thus, these results suggest that HuR and integrin β1/FAK/ERK signaling are needed by Crabp2-mediated promoting effects on metastatic abilities including anoikis resistance, migration, and invasion.

### CRABP2/Crabp2 knockdown has an additive but not synergistic effect on the inhibitory effect of gemcitabine or irinotecan on cell viability

**Figure 6:**
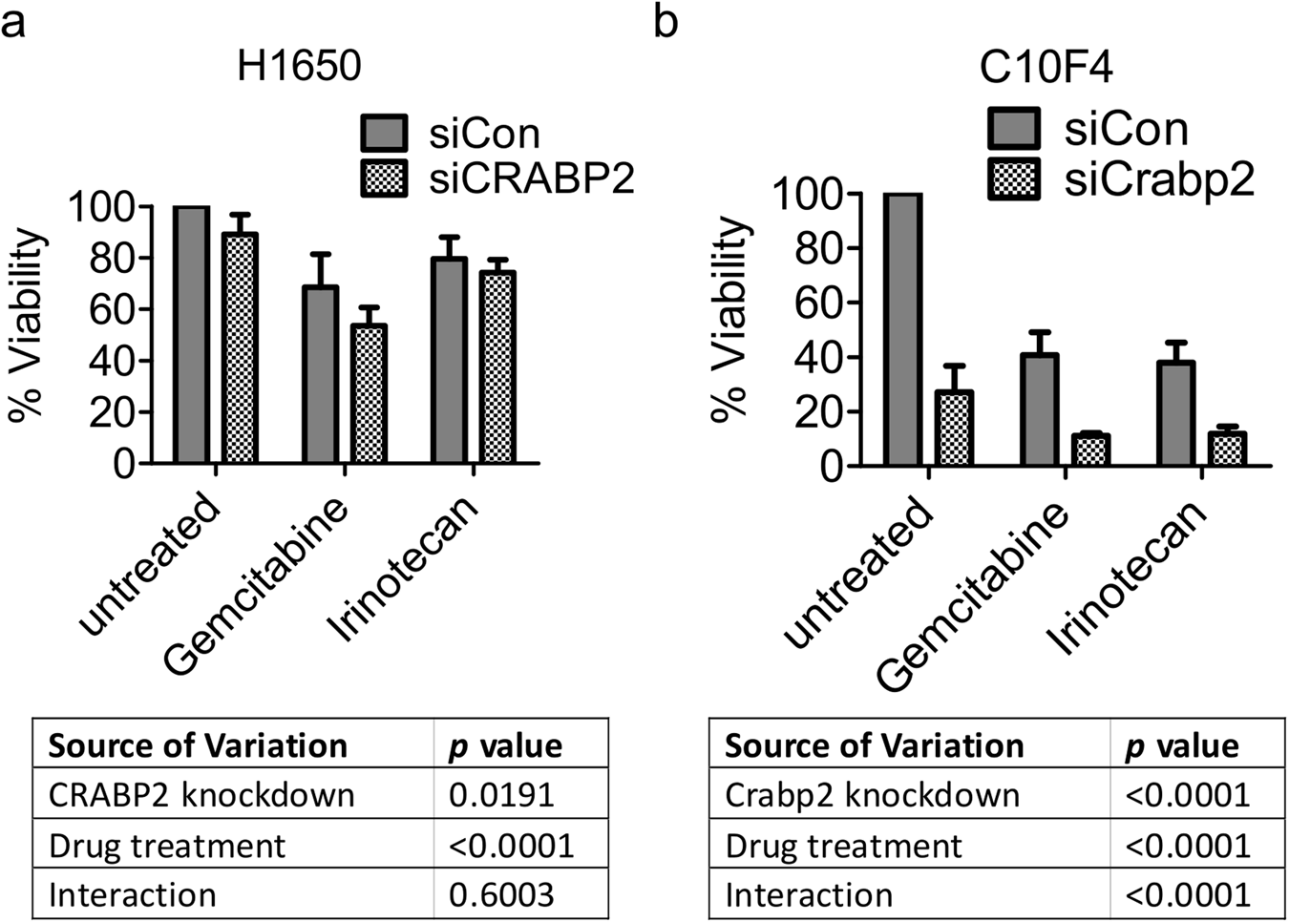
CRABP2/Crabp2 knockdown has an additive but not synergistic effect on the inhibitory effect of gemcitabine and irinotecan on cell viability. (**a**) H1650 cells expressing control-siRNA (siCon) or CRABP2-siRNA (siCRABP2) were plated onto a 96-well plate at a density of 15,000 cells/well. 24 hours later, cells were treated with gemcitabine (10 μM) or etoposide (10 μM). Cell viability was detected by MTS assay at 48 hours after drug treatment. (**b**) C10F4 cells expressing control-siRNA (siCon) or Crabp2-siRNA (siCrabp2) were plated onto a 96-well plate at a density of 15,000 cells/well. 24 hours later, cells were treated with gemcitabine (0.1 μM) or etoposide (50 μM). Cell viability was detected by MTS assay at 72 hours after drug treatment. The *p* values were calculated by Two-way ANOVA.

Erlotinib and gemcitabine are commonly used drugs for non-small cell lung cancer, but their efficiencies are limited as a result of the development of drug resistance^41,42^. Similarly, irinotecan has also been found to show anti-tumor activity in non-small cell lung cancer patients^43^, yet the development of irinotecan resistance has also been reported^44^. We found that elevated expression of CRABP2 was correlated to resistance to seven chemotherapeutics including irinotecan (Table 1), suggesting the potential involvement of CRABP2 in drug resistance. Erlotinib achieved only limited clinical benefits in lung cancer treatment owing to the fact that resistance to erlotinib was found extensively in non-small cell lung cancer patients^42^. Consistently, we found that C10F4 and H1650 cells were not sensitive to erlotinib; treatment with erlotinib at 10 µM did not decrease in the viability of C10F4 and H1650 cells (Fig. S4a,b). In H1650 cells, the combination of CRABP2 knockdown and drug treatment (gemcitabine: 10 µM; irinotecan: 10 µM) resulted in a roughly additive effect of CRABP2 knockdown and drug treatment individually (Fig. 6a), suggesting that CRABP2 and drug treatment have additive but not synergistic effects of inhibition. In C10F4 cells, Crabp2 knockdown or drug treatment alone (gemcitabine: 0.1 µM; irinotecan: 50 µM) caused a nearly 50% inhibition of cell viability (Fig. 6b). The combination of Crabp2 knockdown and drug treatment further reduced cell viability to about 10% (Fig. 6b). These results suggest that Crabp2/CRABP2 knockdown has an additive but not synergistic effect on the inhibitory effect of gemcitabine or irinotecan on cell viability.

**Table 1:** CRABP2 mRNA levels in sensitive and resistant lines of seven chemotherapeutics using data from Barrentina.

Barrentina	n	CRABP2 expression		*p* ^a^
		Low	High	
**CDK Inhibitor PD-0332991**				
Sensitive	13	11	2	0.0110
Resistant	362	175	187	
**Irinotecan**				
Sensitive	71	52	19	0.0002
Resistant	84	36	48	
**MEK Inhibitor PD0325901**				
Sensitive	75	51	24	0.0003
Resistant	311	138	173	
**HDAC Inhibitor Panobinostat**				
Sensitive	86	65	21	<0.0001
Resistant	164	67	97	
**Selumetinib**				
Sensitive	37	28	9	0.0002
Resistant	367	160	207	
**Sorafenib**				
Sensitive	9	8	1	0.0155
Resistant	408	190	218	
**Topotecan**				
Sensitive	154	95	59	0.0002
Resistant	124	48	76	

^a^Fisher’s exact test was used to calculate *p* values.

### Identification of the upstream regulating factor(s) of Crabp2/CRABP2

**Figure 7:**
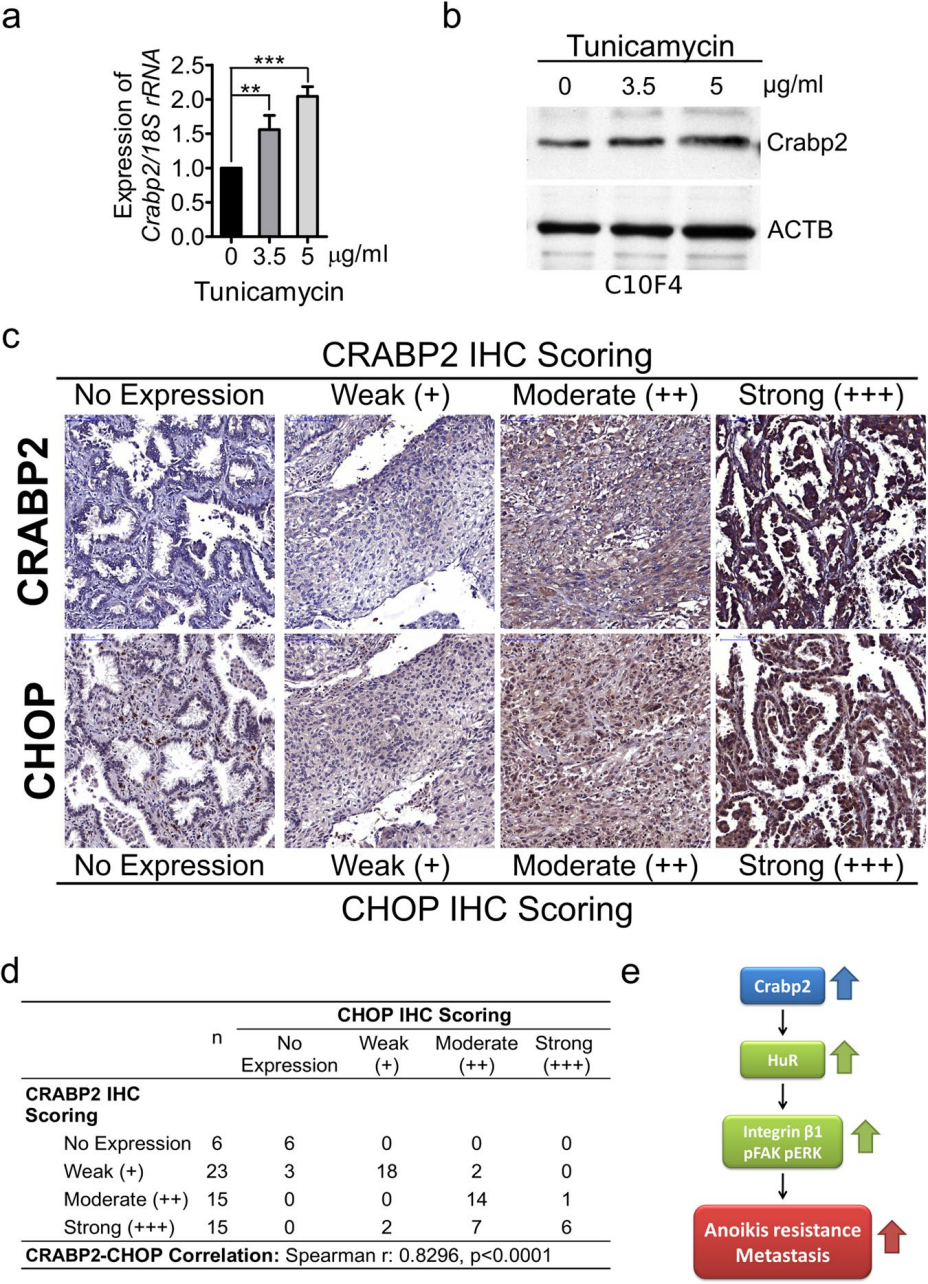
Crabp2 is inducible by tunicamycin and CRABP2 is correlated with CHOP in human lung tumors. (**a**) C10F4 cells were treated with dimethyl sulfoxide (DMSO, as control) or tunicamycin (3.5 or 5 μg/ml) for sixteen hours. Real-time RT-PCR was performed to analyze Crabp2 mRNA levels, and the statistical significance was determined using one-way ANOVA **p < 0.01, ***p < 0.001. (**b**) Western blot of Crabp2 and β-actin (ACTB, as the loading control) from C10F4 cells treated with DMSO (as control) or tunicamycin (3.5 or 5 μg/ml) for sixteen hours. (**c,d**) Immunohistochemical staining of CRABP2 as well as the cell stress marker CHOP in serial-sectioned lung tumor specimens (n = 59) (**c**). The results were assessed and scored by a pathologist, and the correlation between scores of CRABP2 and CHOP was analyzed by Spearman’s correlation test (Spearman r = 0.8299, *p* < 0.0001) (**d**). (**e**) Working hypothesis of Crabp2 regulates HuR and integrin β1/FAK/ERK signaling to promote metastatic behaviors.

As described above, we identified the promoting role of Crabp2 in metastasis of lung cancer cells. However, how Crabp2 is induced in lung tumor cells has never been investigated. To explore the upstream regulator(s) of Crabp2, we performed ontological analysis of genes overexpressed in C10F4 versus C9F6 cells using the Alt-analyze software and identified cellular response to unfolded proteins as one of the most significant biological processes (*p* = 2.35e-3, Z score = 5.3). Unfolded protein response (UPR) is activated in cells when normal functions of the endoplasmic reticulum (ER) are perturbed, a phenomenon termed ER stress^45^. Here we found that treatment with the ER stress inducer tunicamycin^46^ for sixteen hours induced the expression of Crabp2 in C10F4 cells (Fig. 7a,b). We further explored the correlation of CRABP2 with cancer cell stress in human lung tumors. C/EBP homologous protein (CHOP) is a cell stress marker, and is reported to be correlated with lymph node metastasis of non-small cell lung cancer patients^47^. Similar to CRABP2, CHOP is overexpressed in lung tumors (Fig. S5a), and is correlated with poor survival and increased recurrence of lung cancer patients (Fig. S5b,c). We found that CRABP2 levels in human lung tumors were correlated with cell stress marker CHOP (Fig. 7c,d). In another cohort, we also found that CRABP2 levels were correlated with cell stress marker CHOP, E2F1, and E2F7 (Fig. S5d). Thus, the expression of Crabp2 is inducible by tunicamycin-mediated ER stress and is correlated with cell stress markers in lung tumors.

## Discussion

CRABP2 has been found to be overexpressed in multiple cancers including bladder cancer^48^, Wilms tumor^49^, neuroblastoma^50^, pancreatic ductal adenocarcinoma^51^, serous sarcoma^52^, and non-small cell lung cancer (NSCLC)^5–7^. Increased CRABP2 levels were found in patients with metastatic nephroblastomas^53^. In glioblastoma tumors, CRABP2 were found to correlate with poor patient survival^26^. Moreover, CRABP2 promotes survival of malignant peripheral nerve sheath tumors^27^, and it is shown that CRABP2 promotes proliferation of glioblastoma and malignant peripheral nerve sheath tumor cells^26,27^. However, the role of Crabp2 in metastasis of lung cancer has not been investigated until this study. In the present study, we found that Crabp2 was upregulated in lung cancer cells with enhanced metastasis. High CRABP2 levels were correlated with advanced stages, poor overall survival, and recurrence of lung cancer patients. Knockdown of Crabp2 inhibited migration, invasion, anoikis resistance, and *in vivo* metastasis. These results for the first time reveal the promoting effect of Crabp2 in metastatic abilities, and Crabp2 could be a potential prognostic marker for lung cancer patients.

The invasion-metastasis cascade involves multiple cellular capabilities including migration, invasion, and anoikis resistance^8,9^. Moreover, it has been reported that the selected anoikis-resistant pancreatic cancer cells display high migration and invasion ability^11^. Similarly, anoikis-resistant prostate cancer cells show elevated migration and invasion^12^, suggesting that those pro-metastatic abilities are closely-correlated. In the present study, we found that Crabp2 promoted migration, invasion, and anoikis resistance of metastatic lung cancer cells via HuR and integrin β1/FAK/ERK signaling. Our results suggest that Crabp2 may be part of the mechanism regulating migration, invasion, and anoikis resistance in lung cancer cells.

In Fig. 1e, 9 out of 17 C10F4-overexpressing genes were downregulated in tumor versus normal tissues at primary tumor sites. As metastasis is a multi-step process and metastatic tumors reside at different organs with different microenvironment^8^, it is possible that genes promoting the metastatic tumor growth could be different from those for primary tumor growth, or even one gene may inhibit primary tumor growth yet promote metastatic tumor growth due to their different subcellular localization. For instance, connexin 43 (Cx43) in primary tumor cells is known to be localized in the cytoplasm and inhibit tumor growth^54^, whereas in metastatic cancer cells Cx43 is translocated to the cell surface and promotes metastasis via enhancing the adhesion of cancer cells to endothelial cells^55^. Thus, for those 9 genes differentially expressed in C10F4 and primary tumor cells, specific properties of the gene products other than expression levels per se would be relevant to interpret their roles in primary versus metastatic cancer cells.

In Fig. S1, knockdown of Crabp2 reduced the proliferation of C10F4 cells. We also discovered the promoting effect of Crabp2 overexpression on cell proliferation using both MTS assay and trypan blue exclusion assay (Fig. S6). The MTS assay is a colorimetric assay reflecting the number of viable cells with active metabolism^56^. Thus, it is possible that Crabp2 may also be involved in metabolism-related signaling pathways, though further study is needed to elucidate this possibility.

In lung cancer, HuR expression is correlated with lymph node metastasis^32^, and knockdown of HuR inhibited migration and invasion of lung cancer cells^33^. In immortalized breast epithelial cells, knockdown of HuR decreased their anoikis resistance^34^. HuR has been reported in breast cancer to be regulated by Crabp2^29,30^, and here we also found that Crabp2 regulated HuR in lung cancer cells. Integrin β1 is a known downstream of HuR in Jurkat T cells^35^, and integrin signaling molecules including FAK and ERK are critical for migration, invasion, and anoikis resistance of cancer cells^14,16,39,40^. In the present study, we found that Crabp2 activated integrin β1/FAK/ERK signaling via HuR, and thus promoted migration, invasion, and anoikis resistance of metastatic lung cancer cells.

Here we also found that the expression of CRABP2 in human lung tumors was correlated with stress marker CHOP, which was reported to be correlated with lymph node metastasis of non-small cell lung cancer patients^47^. Treatment with ER stress inducer tunicamycin induced Crabp2. ER stress is associated with many diseases including cancer^47^, while its role in tumor progression is still under investigation. Thus, cell stress may be one of the potential factors leading to the upregulation of Crabp2 in lung tumors.

In conclusion, this is the first study to demonstrate the role of Crabp2 in metastasis of lung cancer. Crabp2 promotes migration, invasion, and anoikis resistance via HuR and integrin β1/FAK/ERK signaling (Fig. 7e). Crabp2 might be a potential prognostic biomarker for lung cancer and a therapeutic target to inhibit metastasis and enhance the inhibitory effects of gemcitabine and irinotecan in metastatic lung cancer cells.

## Methods

### Cell culture

The BALB/c lung adenocarcinoma line L1 has been described before^57,58^, and the low-metastatic C9F6 cell line was derived from L1 (unpublished) and was maintained in Dulbecco’s Modified Eagle’s Medium (DMEM; Invitrogen) with 10% FBS (Biological Industries). The H1650 human lung adenocarcinoma cell line was from Dr. Yi Rong Chen of IMGM of NHRI, Taiwan. The authenticity of H1650 cell line was validated by STR profiling service of BCRC (Hsinchu, Taiwan). The H1650 cells were maintained in RPMI 1640 medium (Invitrogen) with 10% FBS.

### Establishment of high-metastatic subline

The C9F6 cells were injected via tail vein at one million cells per mouse. Large nodules were found at lung and were harvested two weeks after injection, and the tumor cells were amplified. This protocol was repeated three times to derive the high-metastatic C10F4 subline.

### RNA extraction and real-time RT-PCR

Detailed procedure was as described^59^. Briefly, total RNA was extracted using TRIzol reagent (Invitrogen), and first-strand cDNA was synthesized using the SuperScript III reverse transcriptase (Invitrogen). SYBR fast and probe fast universal qPCR kits (KAPA) were used for real-time PCR. Taqman probe primer (Thermo, Hs00275636_m1) was used to detect Crabp2 levels in human blood buffy coat samples, and the rest of primers used are described in the Table S6.

### Tissue Samples

The lung cancer cDNA arrays (HLRT101, HLRT103, and HLRT105) were from OriGene. Lung cancer tissue arrays were from Super Bio-Chips (CC5). Blood buffy coat cellular RNAs from 50 healthy people and 49 lung cancer patients were obtained from NRPB lung cancer tissue bank, which obtained informed consent from patients. The experiments were carried out under the National Health Research Institutes Institutional Review Board-approved guidelines (EC1031210-E).

### Exon array

The total RNAs of C9F6, C10F4, and normal mouse lung cells were extracted and analyzed by Affymetrix Mouse Exon 1.0 ST Array (Affymetrix). The result was analyzed by GeneSpring software (Agilent Technologies). The exon array data were uploaded to ArrayExpress (https://www.ebi.ac.uk/arrayexpress) and the accession number is E-MTAB-6512.

### Antibodies and reagents

Detailed information is described in the Table S7.

### Western blot

Detailed procedure was as described^59^. Proteins were visualized using Western Lightning Plus ECL (PerkinElmer). Antibodies used are described in the Table S7.

### Migration and invasion assay

Migration and invasion assays were carried out using 8.0 µm Falcon cell culture insert with or without Matrigel (BD Biosciences) as described^60^. Briefly, 5 * 10^4^ cells were seeded to the upper chambers for the migration and invasion experiments. Cells were photographed and quantified 12 hours (C9F6 and C10F4: migration assay; H1650: migration and invasion assays) or 15 hours (C9F6 and C10F4: invasion assay) later.

### Generation of stable cell lines and plasmid construction

Plasmids and siRNAs were transfected using TransIT-X2 (Mirus). The siRNA for mouse Crabp2 (siCrabp2) was from Dharmacon (Smartpool siGenome Crabp2 siRNA, M-044593-01), and the rest of siRNAs used are described in Table S6. Lentiviral production and infection were performed according to the protocol provided by Sinica RNAi core (Taiwan). Stable cell lines were established by lentiviral infection followed by puromycin selection (Sigma-Aldrich) or fluorescence-activated cell sorting (FACS). For Crabp2 expression plasmid, the mouse Crabp2 was amplified from cDNAs of C10F4 cells. The shRNA lentiviral plasmid for mouse Crabp2 (TRCN0000105235), HuR (TRCN0000308993), and RFP (TRCN0000072203, as control shRNA) were from Sinica RNAi core (Taiwan).

### Anoikis assay

Cell viability under loss of attachment was measured using CytoSelect anoikis assay kit (Cell Biolabs) following the manufacturer’s instruction. Briefly, cells were plated onto a 96-well anchorage-resistant plate (Cell Biolabs) at a density of 10,000 cells/well. Twenty-four hours later, the viability of cells was assessed by MTS assay (Promega).

### Tail vein metastasis assay

6- to 8-weeks-old BALB/c mice were from National Laboratory Animal Center (Taiwan) and were randomized into experimental groups before implantation of tumor cells. One million cells were suspended in 100 µl PBS, and then injected into mouse tail vein, and mouse organs were harvested after cardiac perfusion with PBS. Mouse organs were fixed with paraformaldehyde, paraffin-embedded, and sectioned for hematoxylin and eosin (H&E) staining. For quantification of GFP-expressing cancer cells, mouse organs were subjected to RNA extraction for detection of GFP RNAs by real-time RT-PCR. The BALB/c mice were provided by the National Laboratory Animal Center (Taiwan). All animal studies were approved by Institutional Animal Care and Use Committee (IACUC) of National Health Research Institutes and all methods were carried out in accordance with the relevant guidelines and regulations (NHRI-IACUC-105127).

### IHC staining and analysis

IHC staining was performed using an automatic slide stainer BenchMark XT (Ventana Medical Systems). IHC examination and the scoring report was done by a pathologist of the Pathology Core Laboratory of National Health Research Institutes.

### Cell viability assays

Cells were plated onto a 96-well plate at a density of 15,000 cells/well. Twenty-four hours later, cells were treated with dimethyl sulfoxide (DMSO), gemcitabine (Sigma), erlotinib (Selleckchem), or irinotecan (Selleckchem). Forty-eight (H1650) or seventy-two (C10F4) hours later, cell viability was assessed by MTS assay (Promega).

### Immunoprecipitation

The Crabp2 antibody (Proteintech) was crosslinked to protein G-agarose beads (Millipore) by disuccinimidyl suberate (DSS, Thermo). The rest of the procedure was as described^59^. Briefly, cell lysates (500 µg) were incubated with resin-antibody complex 1 hour at 4 °C. Protein-antibody complexes were then washed using lysis buffer. Denatured proteins were analyzed by Western blotting.

### Statistical analysis

Data of triplicate experiments were analyzed using GraphPad Prism (GraphPad Software Inc, San Diego, CA) and presented as mean ± SD (standard deviation). Differences between two groups were analyzed using Student’s t test. Differences among more than two groups were analyzed using one-way ANOVA and Tukey’s test as post test. For tissue samples, differences between two groups were analyzed using Mann-Whitney test, and differences between more than two groups were analyzed using Kruskal-Wallis test and Dunn’s test as post test. The correlation between two variables was analyzed using Spearman’s correlation test. Frequency distributions between categorical variables were compared using Fisher’s exact test (for 2 × 2) and chi-square test. Differences were considered significant at *p* < 0.05 (*p < 0.05, **p < 0.01 and ***p < 0.001), and ns represents no significance.

## Supplementary information

Supplementary Information

## References

[1] Duggan MA, Anderson WF, Altekruse S, Penberthy L, Sherman ME (2016). The Surveillance, Epidemiology, and End Results (SEER) Program and Pathology: Toward Strengthening the Critical Relationship. Am. J. Surg. Pathol..

[2] Donovan M, Olofsson B, Gustafson AL, Dencker L, Eriksson U (1995). The cellular retinoic acid binding proteins. J. Steroid Biochem. Mol. Biol..

[3] Kainov Y (2014). CRABP1 provides high malignancy of transformed mesenchymal cells and contributes to the pathogenesis of mesenchymal and neuroendocrine tumors. Cell Cycle.

[4] Liu R-Z (2015). CRABP1 is associated with a poor prognosis in breast cancer: adding to the complexity of breast cancer cell response to retinoic acid. Mol. Cancer.

[5] Han S-S (2014). RNA sequencing identifies novel markers of non-small cell lung cancer. Lung Cancer.

[6] Codreanu SG (2017). Identification of Proteomic Features To Distinguish Benign Pulmonary Nodules from Lung Adenocarcinoma. J. Proteome Res..

[7] Zhang Y (2015). Global analysis of chromosome 1 genes among patients with lung adenocarcinoma, squamous carcinoma, large-cell carcinoma, small-cell carcinoma, or non-cancer. Cancer Metastasis Rev..

[8] Valastyan S, Weinberg RA (2011). Tumor metastasis: molecular insights and evolving paradigms. Cell.

[9] Kim Y-N, Koo KH, Sung JY, Yun U-J, Kim H (2012). Anoikis resistance: an essential prerequisite for tumor metastasis. Int. J. Cell Biol..

[10] Taddei ML, Giannoni E, Fiaschi T, Chiarugi P (2012). Anoikis: an emerging hallmark in health and diseases. J. Pathol..

[11] Fofaria NM, Srivastava SK (2015). STAT3 induces anoikis resistance, promotes cell invasion and metastatic potential in pancreatic cancer cells. Carcinogenesis.

[12] Zhang, P. *et al*. AMPK/GSK3β/β-catenin cascade-triggered overexpression of CEMIP promotes migration and invasion in anoikis-resistant prostate cancer cells by enhancing metabolic reprogramming. *FASEB J*. fj201701078R (2018). 10.1096/fj.201701078R 29505302

[13] Tai Y-L, Chen L-C, Shen T-L (2015). Emerging roles of focal adhesion kinase in cancer. Biomed Res. Int..

[14] Frisch SM, Vuori K, Ruoslahti E, Chan-Hui PY (1996). Control of adhesion-dependent cell survival by focal adhesion kinase. J. Cell Biol..

[15] Pachmayr E, Treese C, Stein U (2017). Underlying Mechanisms for Distant Metastasis - Molecular Biology. Visc Med.

[16] Collins NL (2005). G1/S cell cycle arrest provides anoikis resistance through Erk-mediated Bim suppression. Mol. Cell. Biol..

[17] Golubovskaya VM (2010). Focal adhesion kinase as a cancer therapy target. Anticancer Agents Med. Chem..

[18] Kohno M, Pouyssegur J (2006). Targeting the ERK signaling pathway in cancer therapy. Ann. Med..

[19] Roh ME, Cosgrove M, Gorski K, Hitchcock IS (2013). Off-targets effects underlie the inhibitory effect of FAK inhibitors on platelet activation: studies using Fak-deficient mice. J. Thromb. Haemost..

[20] Rhodes DR (2004). ONCOMINE: a cancer microarray database and integrated data-mining platform. Neoplasia.

[21] Fidler IJ, Kripke ML (2015). The challenge of targeting metastasis. Cancer Metastasis Rev..

[22] Uramoto H, Tanaka F (2014). Recurrence after surgery in patients with NSCLC. Transl Lung Cancer Res.

[23] Győrffy B, Surowiak P, Budczies J, Lánczky A (2013). Online survival analysis software to assess the prognostic value of biomarkers using transcriptomic data in non-small-cell lung cancer. PLoS One.

[24] Paesmans M (2012). Prognostic and predictive factors for lung cancer. Breathe.

[25] Coate LE, John T, Tsao M-S, Shepherd FA (2009). Molecular predictive and prognostic markers in non-small-cell lung cancer. Lancet Oncol..

[26] Liu R-Z (2016). Association between cytoplasmic CRABP2, altered retinoic acid signaling, and poor prognosis in glioblastoma. Glia.

[27] Fischer-Huchzermeyer S (2017). The Cellular Retinoic Acid Binding Protein 2 Promotes Survival of Malignant Peripheral Nerve Sheath Tumor Cells. Am. J. Pathol..

[28] Mohammad Sultan, K. M. C. Retinoid Signaling in Cancer and Its Promise for Therapy. *J Carcinog Mutagen*, 10.4172/2157-2518.S7-006 (2013).

[29] Vreeland AC, Levi L, Zhang W, Berry DC, Noy N (2014). Cellular retinoic acid-binding protein 2 inhibits tumor growth by two distinct mechanisms. J. Biol. Chem..

[30] Vreeland AC, Yu S, Levi L, de Barros Rossetto D, Noy N (2014). Transcript stabilization by the RNA-binding protein HuR is regulated by cellular retinoic acid-binding protein 2. Mol. Cell. Biol..

[31] Wang J (2013). Multiple functions of the RNA-binding protein HuR in cancer progression, treatment responses and prognosis. Int. J. Mol. Sci..

[32] Wang J (2009). The expression of RNA-binding protein HuR in non-small cell lung cancer correlates with vascular endothelial growth factor-C expression and lymph node metastasis. Oncology.

[33] Muralidharan R (2015). HuR-targeted nanotherapy in combination with AMD3100 suppresses CXCR4 expression, cell growth, migration and invasion in lung cancer. Cancer Gene Ther..

[34] Heinonen M (2011). Role of RNA binding protein HuR in ductal carcinoma *in situ* of the breast. J. Pathol..

[35] Mukherjee N, Lager PJ, Friedersdorf MB, Thompson MA, Keene JD (2009). Coordinated posttranscriptional mRNA population dynamics during T-cell activation. Mol. Syst. Biol..

[36] Teng Y-C (2013). Histone demethylase RBP2 promotes lung tumorigenesis and cancer metastasis. Cancer Res..

[37] Ganguly KK, Pal S, Moulik S, Chatterjee A (2013). Integrins and metastasis. Cell Adh. Migr..

[38] Sulzmaier FJ, Jean C, Schlaepfer DD (2014). FAK in cancer: mechanistic findings and clinical applications. Nat. Rev. Cancer.

[39] Miyazaki T (2003). FAK overexpression is correlated with tumour invasiveness and lymph node metastasis in oesophageal squamous cell carcinoma. Br. J. Cancer.

[40] Guo W, Giancotti FG (2004). Integrin signalling during tumour progression. Nat. Rev. Mol. Cell Biol..

[41] Rosell R (2003). Targeted therapy in combination with gemcitabine in non-small cell lung cancer. Semin. Oncol..

[42] Lin Y, Wang X, Jin H (2014). EGFR-TKI resistance in NSCLC patients: mechanisms and strategies. Am. J. Cancer Res..

[43] Wills B (2017). Survival Outcomes According to TIMP1 and EGFR Expression in Heavily Treated Patients with Advanced Non-small Cell Lung Cancer who Received Biweekly Irinotecan Plus Bevacizumab. Anticancer Res..

[44] Xu Y, Villalona-Calero MA (2002). Irinotecan: mechanisms of tumor resistance and novel strategies for modulating its activity. Ann. Oncol..

[45] Wang W-A, Groenendyk J, Michalak M (2014). Endoplasmic reticulum stress associated responses in cancer. Biochim. Biophys. Acta.

[46] Lee AS (2001). The glucose-regulated proteins: stress induction and clinical applications. Trends Biochem. Sci..

[47] Xu Y (2016). HSP90B1 overexpression predicts poor prognosis in NSCLC patients. Tumour Biol..

[48] Jin B-Y (2013). CRABP2 and FABP5 identified by 2D DIGE profiling are upregulated in human bladder cancer. Chin. Med. J..

[49] Gupta A, Kessler P, Rawwas J, Williams BRG (2008). Regulation of CRABP-II expression by MycN in Wilms tumor. Exp. Cell Res..

[50] Gupta A, Williams BRG, Hanash SM, Rawwas J (2006). Cellular retinoic acid-binding protein II is a direct transcriptional target of MycN in neuroblastoma. Cancer Res..

[51] Xiao W (2014). CRABP-II is a highly sensitive and specific diagnostic molecular marker for pancreatic ductal adenocarcinoma in distinguishing from benign pancreatic conditions. Hum. Pathol..

[52] Toyama A (2012). Proteomic characterization of ovarian cancers identifying annexin-A4, phosphoserine aminotransferase, cellular retinoic acid-binding protein 2, and serpin B5 as histology-specific biomarkers. Cancer Sci..

[53] Percicote AP (2018). Tissue expression of retinoic acid receptor alpha and CRABP2 in metastatic nephroblastomas. Diagn. Pathol..

[54] Xu H-T (2008). Connexin 43 recruits E-cadherin expression and inhibits the malignant behaviour of lung cancer cells. Folia Histochem. Cytobiol..

[55] Stoletov K (2013). Role of connexins in metastatic breast cancer and melanoma brain colonization. J. Cell Sci..

[56] Cory AH, Owen TC, Barltrop JA, Cory JG (1991). Use of an aqueous soluble tetrazolium/formazan assay for cell growth assays in culture. Cancer Commun..

[57] Sun AS (2001). Pilot study of a specific dietary supplement in tumor-bearing mice and in stage IIIB and IV non-small cell lung cancer patients. Nutr. Cancer.

[58] Bahler DW, Frelinger JG, Harwell LW, Lord EM (1987). Reduced tumorigenicity of a spontaneous mouse lung carcinoma following H-2 gene transfection. Proc. Natl. Acad. Sci. USA.

[59] Lin K-T (2012). Vav3-rac1 signaling regulates prostate cancer metastasis with elevated Vav3 expression correlating with prostate cancer progression and posttreatment recurrence. Cancer Res..

[60] Yeh Y-M, Chuang C-M, Chao K-C, Wang L-H (2013). MicroRNA-138 suppresses ovarian cancer cell invasion and metastasis by targeting SOX4 and HIF-1α. Int. J. Cancer.

