# Supplementary material for: Crabp2 Promotes Metastasis of Lung Cancer Cells via HuR and Integrin β1/FAK/ERK Signaling
Source: Sci Rep. 2019 Jan 29;9:845. doi: 10.1038/s41598-018-37443-4 (PMC6351595; doi:10.1038/s41598-018-37443-4)

Supplementary Information

Crabp2 Promotes Metastasis of Lung Cancer Cells via HuR and Integrin β1/FAK/ERK Signaling

Jun-I Wu[1, 2, 6], Yi-Pei Lin[1, 3, 6], Chien-Wei Tseng[1, 4], Hui-Jane Chen[1] and Lu-Hai Wang[1, 2, 3, 4, 5*]

[1]Institute of Molecular and Genomic Medicine, National Health Research Institutes, Miaoli County, Taiwan

[2] Department of Life Sciences, National Central University, Taoyuan, Taiwan

[3]Institute of Molecular and Cellular Biology, National Taiwan University, Taipei, Taiwan

[4]Graduate Institute of Integrated Medicine, China Medical University, Taichung, Taiwan

[5]Chinese Medical Research Center, China Medical University, Taichung, Taiwan

[6]These authors contributed equally to this work

*Correspondence and requests for materials should be addressed to: Dr. Lu-Hai Wang, Graduate Institute of Integrated Medicine, China Medical University, Taichung, Taiwan. E-mail: lu-hai.wang@nhri.org.tw or luhaiwang@mail.cmu.edu.tw. TEL: 886 4-2205 715; FAX: 886 4-2206 0248

Supplementary Figure Legends

Figure S1. Knockdown of Crabp2 inhibited proliferation of C10F4 cells. C10F4 cells expressing control-shRNA (shCon) or Crabp2 shRNA (shCrabp2) were plated onto a 96-well plate at a density of 5,000 cells/well. Cell abundance was detected at 1-4 days by MTS assay. The *p* values were determined using Student's t test **p*<0.05, ****p*<0.001.

Figure S2. The inhibitory effect of retinoic acid on anoikis resistance of C10F4 cells is independent of Crabp2. C10F4 cells expressing empty vector or Crabp2 were plated onto a 96-well anchorage-resistant plate at a density of 10,000 cells/well and treated with dimethyl sulfoxide (DMSO) or retinoic acid (RA, 1/10/50 μM). Twenty-four hours later, the viability of cells treated with RA versus DMSO was assessed by MTS assay. The *p* values were determined using two-way ANOVA.

Figure S3. Knockdown of HuR expression. Western blot analysis of HuR and β-actin (ACTB, as the loading control) in C10F4 cells expressing control-shRNA (shCon) or HuR-shRNA (shHuR).

Figure S4. C10F4 and H1650 cells are not sensitive to erlotinib. (a-b) C10F4 (a) or H1650 (b) cells were exposed to 10 μM erlotinib. Cell viability at 48 hours was detected by MTS assay. The *p* values were calculated by Student's t test, and ns for *p*>0.05.

Figure S5. CRABP2 is correlated with cell stress marker CHOP, E2F1, and E2F7. (a) Levels of CRABP2 and cell stress marker CHOP, E2F1, and E2F7 in tumors (T,

n=91) versus normal lungs (N, n=65) are shown using data from Hou lung, and the statistical significance was determined using Kruskal-Wallis test and Dunn's test as post test ***p*<0.01, ****p*<0.001. (b-c) Kaplan-Meier plot of the overall survival of 1926 lung cancer patients (b), or the first progression after surgery of 982 lung cancer patients (c) stratified by expression levels of CHOP using data from Kaplan-Meier Plotter database. The optimal cutoff was derived from Kaplan-Meier Plotter database, and patients with high levels of CHOP ($CHOP^{High}$) exhibited higher risk than patients with low levels CHOP ($CHOP^{Low}$). (d) Scatter plots of mRNA levels of CHOP (left), E2F1 (middle), or E2F7 (right) (as X-axes) versus CRABP2 (as Y-axis) in lung tumors and normal lungs using data from Hou lung (n=156). The correlation of expression between CRABP2 and CHOP (Spearman r=0.3109, *p*<0.0001), E2F1 (Spearman r=0.4796, *p*<0.0001), or E2F7 (Spearman r=0.5886, *p*<0.0001) was analyzed by Spearman's correlation test.

Figure S6. Overexpression of Crabp2 promoted proliferation of C10F4 cells. (a) C10F4 cells expressing empty vector (Vector) or Crabp2 were plated onto a 96-well plate at a density of 1,000 cells/well. Cell abundance was detected at 1-4 days by MTS assay. (b) C10F4 cells expressing empty vector (Vector) or Crabp2 were plated onto a 6-well plate at a density of $4*10^5$ cells/well. Cell abundance was detected at 1-3 days by trypan blue exclusion assay using TC20 automated cell counter. The *p* values were determined using Student's t test **p*<0.05, ***p*<0.01.

Table S1. Correlation of CRABP2 expression levels with clinicopathologic features of lung tumors.

HLRT[a]	n	CRABP2 expression		*p*
		Low	High	
**Gender**				
Female	45	27	18	0.1310[b]
Male	74	33	41	
**Age**				
≤70	73	40	33	0.2617[b]
>70	47	20	27	
**Lung Cancer Type**				
NSCLC	115	55	60	0.0573[b]
SCLC	5	5	0	
**NSCLC Subtype**				
Adenocarcinoma	48	24	24	0.6052[c]
Squamous cell carcinoma	40	16	24	
Large cell carcinoma	10	6	4	
Non-small cell	17	9	8	
**Stage**				
I	43	24	19	0.5334[c]
II	37	16	21	
III-IV	40	20	20	

CRABP2 levels in tumor samples (n=120) were detected by real-time PCR. Patients in individual clinical factors were stratified according to low and high CRABP2 levels based on the median value. NSCLC: non-small cell lung cancer; SCLC: small cell lung cancer. [a]HLRT: human lung cancer cDNA array. [b]Fisher's exact test was used to calculate *p* values. [c]χ^2 test was used to calculate *p* values.

Table S2. Multivariate survival analysis of CRABP2 in lung cancer.

	Overall survival[a]		First progression[b]	
	p value	HR (95% CI)	*p* value	HR (95% CI)
Histology	<0.0001	1.44 (1.22-1.7)	0.7332	0.93 (0.63-1.39)
Stage	<0.0001	1.52 (1.33-1.73)	<0.0001	2.21 (1.71-2.86)
Gender	0.0036	1.37 (1.11-1.68)	0.0725	1.35 (0.97-1.88)
CRABP2	0.0306	1.31 (1.03-1.67)	0.0074	1.71 (1.15-2.54)

[a]Multi-variate analysis (Cox regression) of the overall survival of 890 lung cancer patients. [b]Multi-variate analysis (Cox regression) of the first progression after surgery of 469 lung cancer patients. The optimal cutoff, *p* values and hazard ratio (HR) were provided by Kaplan-Meier Plotter database.

Table S3. Multivariate survival analysis of ERCC1 in lung cancer.

	Overall survival[a]		First progression[b]	
	p value	HR (95% CI)	*p* value	HR (95% CI)
Histology	0.0001	1.4 (1.19-1.65)	0.6234	0.9 (0.61-1.35)
Stage	<0.0001	1.56 (1.36-1.78)	<0.0001	2.27 (1.75-2.94)
Gender	0.0086	1.32 (1.07-1.63)	0.1297	1.29 (0.93-1.79)
ERCC1	0.001	1.41 (1.15-1.74)	0.3838	0.85 (0.59-1.22)

[a]Multi-variate analysis (Cox regression) of the overall survival of 890 lung cancer patients. [b]Multi-variate analysis (Cox regression) of the first progression after surgery of 469 lung cancer patients. The optimal cutoff, *p* values and hazard ratio (HR) were provided by Kaplan-Meier Plotter database.

Table S4. Multivariate survival analysis of p27 in lung cancer.

	Overall survival[a]		First progression[b]	
	p value	HR (95% CI)	*p* value	HR (95% CI)
Histology	0.0004	1.35 (1.15-1.6)	0.8263	0.96 (0.64-1.43)
Stage	<0.0001	1.52 (1.34-1.74)	<0.0001	2.29 (1.78-2.96)
Gender	0.0056	1.35 (1.09-1.66)	0.1509	1.27 (0.92-1.77)
p27	<0.0001	1.64 (1.3-2.07)	0.1606	0.79 (0.57-1.1)

[a]Multi-variate analysis (Cox regression) of the overall survival of 890 lung cancer patients. [b]Multi-variate analysis (Cox regression) of the first progression after surgery of 469 lung cancer patients. The optimal cutoff, *p* values and hazard ratio (HR) were provided by Kaplan-Meier Plotter database.

Table S5. Multivariate survival analysis of RRM1 in lung cancer.

	Overall survival[a]		First progression[b]	
	p value	HR (95% CI)	*p* value	HR (95% CI)
Histology	<0.0001	1.52 (1.29-1.8)	0.3598	0.83 (0.55-1.24)
Stage	<0.0001	1.56 (1.36-1.78)	<0.0001	2.2 (1.68-2.87)
Gender	0.0074	1.33 (1.08-1.64)	0.1359	1.28 (0.92-1.78)
RRM1	0.0001	0.63 (0.5-0.79)	0.0194	1.53 (1.07-2.18)

[a]Multi-variate analysis (Cox regression) of the overall survival of 890 lung cancer patients. [b]Multi-variate analysis (Cox regression) of the first progression after surgery of 469 lung cancer patients. The optimal cutoff, *p* values and hazard ratio (HR) were provided by Kaplan-Meier Plotter database.

Table S6. The list of primers and oligomers used in this study.

Oligomers	Sequences
18S rRNA primers with Taqman probes*,[a]	Forward: 5'-GCATGGCCGTTCTTAGTTGG-3'<br>Reverse: 5'-TGCCAGAGTCTCGTTCGTTA-3'<br>Probe: 5'-TGGAGCGATTTGTCTGGTTAATTCCGA-3'
GFP primers with Taqman probes*	Forward: 5'-ACTACCTGAGCACCCAGTCC-3'<br>Reverse: 5'-CTTGTACAGCTCGTCCATGC-3'<br>Probe: 5'-CCCAACGAGAAGCGCGATCA-3'
18S rRNA	Forward: 5'-GTAACCCGTTGAACCCCATT-3'<br>Reverse: 5'-CCATCCAATCGGTAGTAGCG-3'
Mouse Crabp2	Forward: 5'-ATGCCTAACTTTTCTGGCAACT-3'<br>Reverse: 5'-CCTGTTTGATCTCGACTGCTG-3'
Mouse HuR	Forward: 5'-CATCCCTACCACCCAACACC-3'<br>Reverse: 5'-CCAGACAGTGGAGACCACAC-3'
Mouse ITGB1 (integrin β1)	Forward: 5'-TAGACATGTGGGGAGTGGGT-3'<br>Reverse: 5'-AGTTCTCCAGCATCTAGTAGCC-3'
Human CRABP2	Forward: 5'-GATGCCTCTTGCAGGGTCTT-3'<br>Reverse: 5'-GTGAACCCGGAATGGGTGAT-3'
Control shRNA (RFP, TRCN0000072203)	5'-CCGGCGCGTGATGAACTTCGAGGACCTCGAGGTCCTCGAAGTTCATCACGCGTTTTTG-3'
Mouse Crabp2 shRNA (TRCN0000105235)	5'-CCGGCCACGTCCATCTTACAAACTACTCGAGTAGTTTGTAAGATGGACGTGGTTTTTG-3'
Mouse HuR shRNA (TRCN0000308993)	5'-CCGGCATTGGGAGAACGAATTTAATCTCGAGATTAAATTCGTTCTCCCAATGTTTTTG-3'

Control siRNA	Sense: 5'-UUCUCCGAACGUGUCACGUTT-3'<br>Anti-sense: 3'-TTAAGAGGCUUGCACAGUGCA-5'
Human CRABP2 siRNA	Sense: 5'-CGGAAAACUUCGAGGAAUU-3'<br>Anti-sense: 3'-TTGCCUUUUGAAGCUCCUUAA-5'
Mouse HuR siRNA (A)	Sense: 5'-GGAGAACGAAUUUAAUUGUTT-3'<br>Anti-sense: 3'-TTCCUCUUGCUUAAAUUAACA-5'
Mouse HuR siRNA (B)	Sense: 5'-GCUGGUGCAUCUUCAUCUATT-3'<br>Anti-sense: 3'-TTCGACCACGUAGAAGUAGAU-5'
Mouse HuR siRNA (C)	Sense: 5'-GACCAUGACAAACUAUGAATT-3'<br>Anti-sense: 3'-TTCUGGUACUGUUUGAUACUU-5'
Mouse Integrin β1 siRNA (A)	Sense: 5'-GGCUCUCAAACUAUAAAGATT-3'<br>Anti-sense: 3'-TTCCGAGAGUUUGAUAUUUCU-5'
Mouse Integrin β1 siRNA (B)	Sense: 5'-GGGCUGAAGAUUACCCUAUTT-3'<br>Anti-sense: 3'-TTCCCGACUUCUAAUGGGAUA-5'
Mouse Integrin β1 siRNA (C)	Sense: 5'-GCGAGUGUGAUAACUUCAATT-3'<br>Anti-sense: 3'-TTCGCUCACACUAUUGAAGUU-5'

*: used for detection of GFP+ cells in mouse tissues. [a]: used for real-time PCR of blood buffy coat samples.

Table S7. Information of antibodies and reagents used in this study.

**Western blot antibodies**	
β-actin	Genetex, GTX109639
CRABP2	Genetex, GTX101551
HuR	Cell signaling, 12582
Integrin β1	Santa cruz, sc-6622
pERK (T202/Y204)	Cell signaling, 9101
ERK	Cell signaling, 9102
pFAK (Y397)	Genetex, GTX61795
FAK	Millipore, 05-537
**Immunoprecipitation antibodies**	
CRABP2	Proteintech, 10225-1-AP
**IHC antibodies**	
CRABP2	Genetex, GTX101551
CHOP	Genetex, GTX11419
**Chemicals**	
Tunicamycin	Sigma, T7765
Gemcitabine	Sigma, G6423
Erlotinib	Selleckchem, S7786
Irinotecan	Selleckchem, S2217
Retinoic acid	Sigma, R2625
ERK inhibitor FR180204	Sigma, SML0320

FAK inhibitor 14	Sigma, SML0837

Figure S1

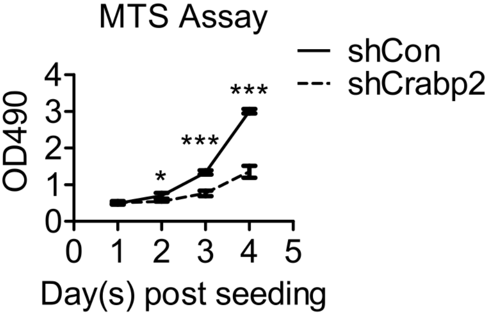
MTS Assay
shCon
shCrabp2
OD490
0
1
2
3
4
*
***
***
0
1
2
3
4
5
Day(s) post seeding

Figure S2

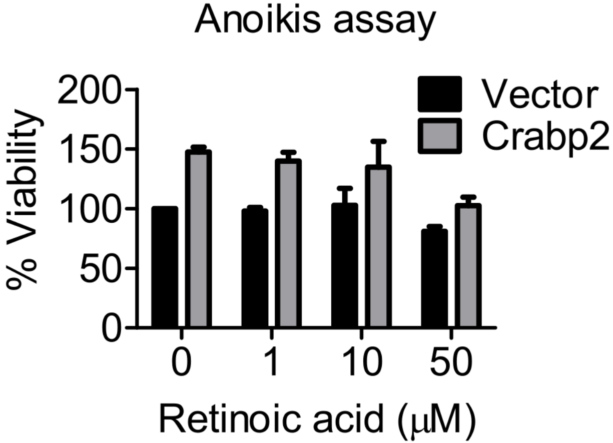

**Source of Variation**	***p* value**
Crabp2 overexpression	<0.0001
Retinoic acid	0.0002
Interaction	0.1677

Figure S3

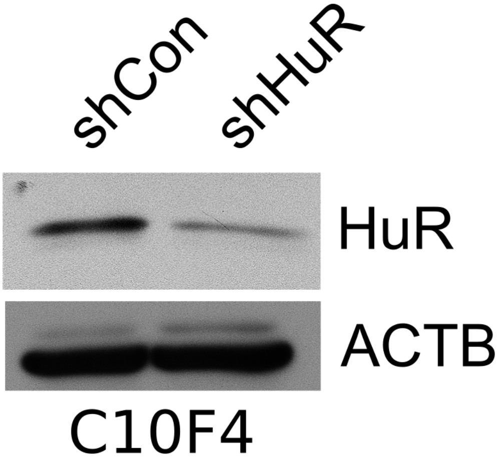

Figure S4

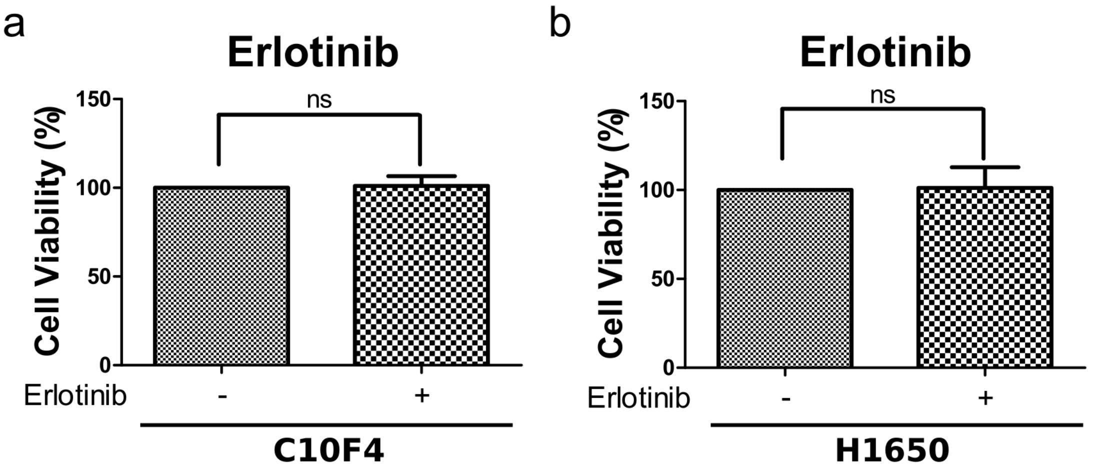
a
Erlotinib
Cell Viability (%)
150
100
50
0
ns
Erlotinib
-
+
C10F4
b
Erlotinib
Cell Viability (%)
150
100
50
0
ns
Erlotinib
-
+
H1650

Figure S5

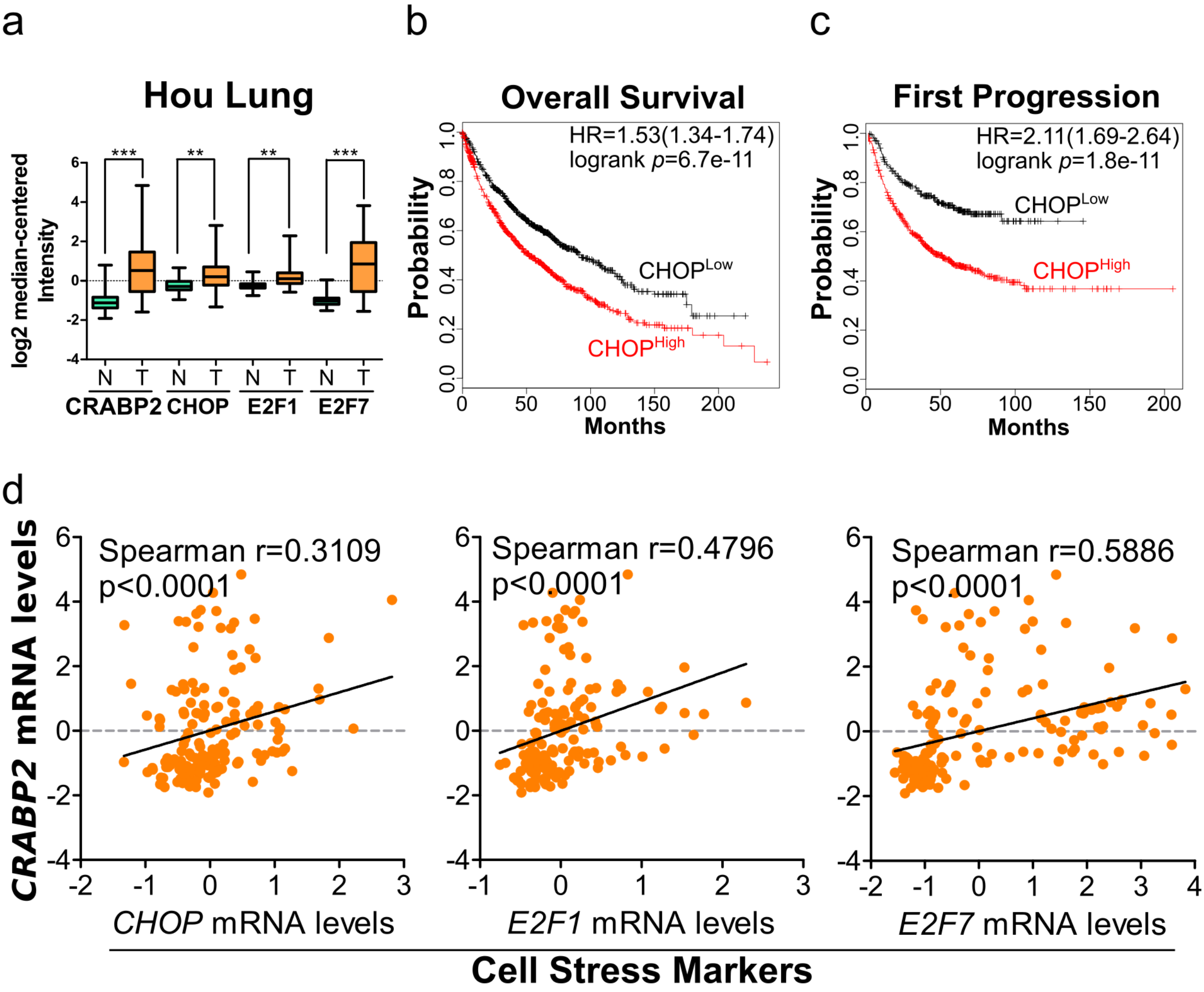
a
Hou Lung
log2 median-centered Intensity
***
**
**
***
N T N T N T N T
CRABP2 CHOP E2F1 E2F7
b
Overall Survival
HR=1.53(1.34-1.74)
logrank p=6.7e-11
CHOPLow
CHOPHigh
Probability
Months
c
First Progression
HR=2.11(1.69-2.64)
logrank p=1.8e-11
CHOPLow
CHOPHigh
Probability
Months
d
CRABP2 mRNA levels
Spearman r=0.3109
p<0.0001
CHOP mRNA levels
Spearman r=0.4796
p<0.0001
E2F1 mRNA levels
Spearman r=0.5886
p<0.0001
E2F7 mRNA levels
Cell Stress Markers

Figure S6

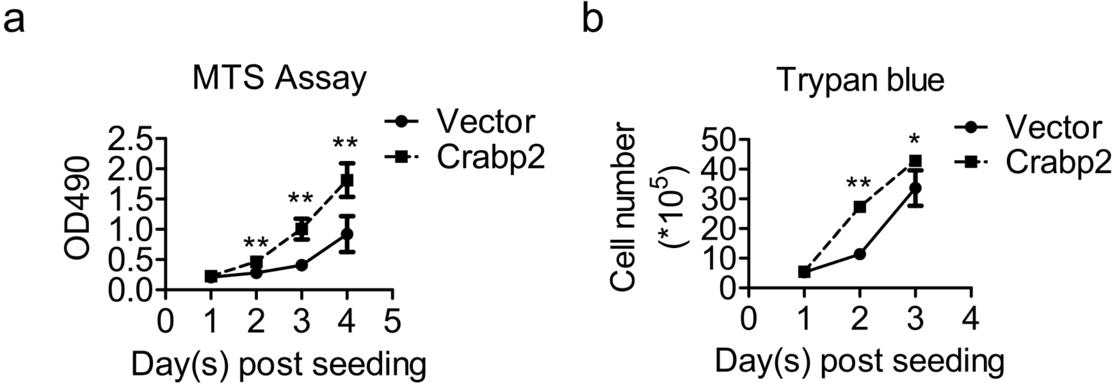
a
MTS Assay
Vector
Crabp2
OD490
2.5
2.0
1.5
1.0
0.5
0.0
**
**
**
0
1
2
3
4
5
Day(s) post seeding
b
Trypan blue
Vector
Crabp2
Cell number
(*10^5)
50
40
30
20
10
0
*
**
0
1
2
3
4
Day(s) post seeding

Figure 1g

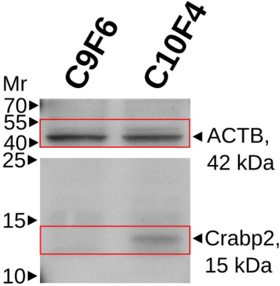

Figure 3a

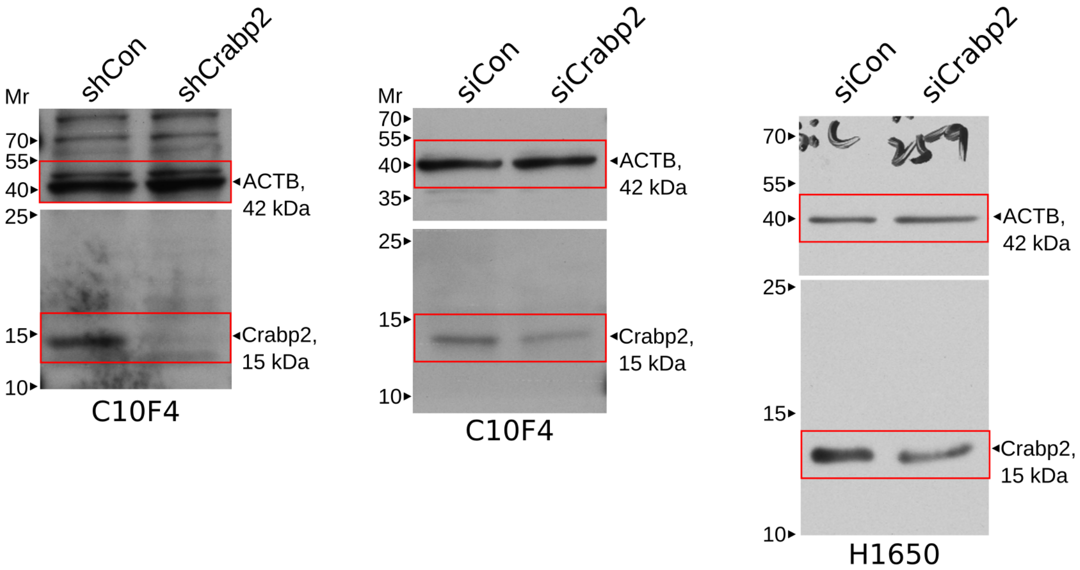

shCon
shCrabp2
Mr
70
55
40
25
15
10
ACTB, 42 kDa
Crabp2, 15 kDa
C10F4
siCon
siCrabp2
Mr
70
55
40
35
25
15
10
ACTB, 42 kDa
Crabp2, 15 kDa
C10F4
siCon
siCrabp2
70
55
40
25
15
10
ACTB, 42 kDa
Crabp2, 15 kDa
H1650

Figure 4a

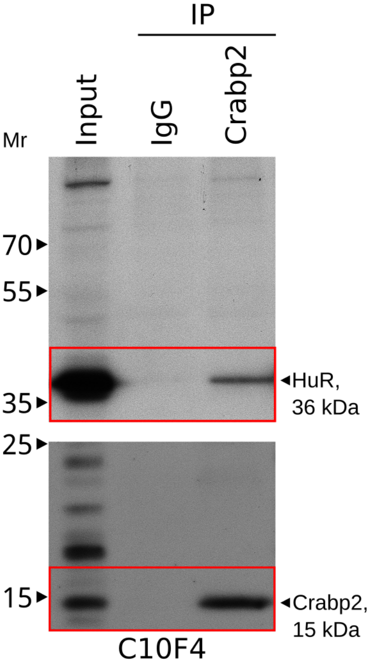
IP
Mr
Input
IgG
Crabp2
70
55
35
25
15
HuR,
36 kDa
Crabp2,
15 kDa
C10F4

Figure 4f

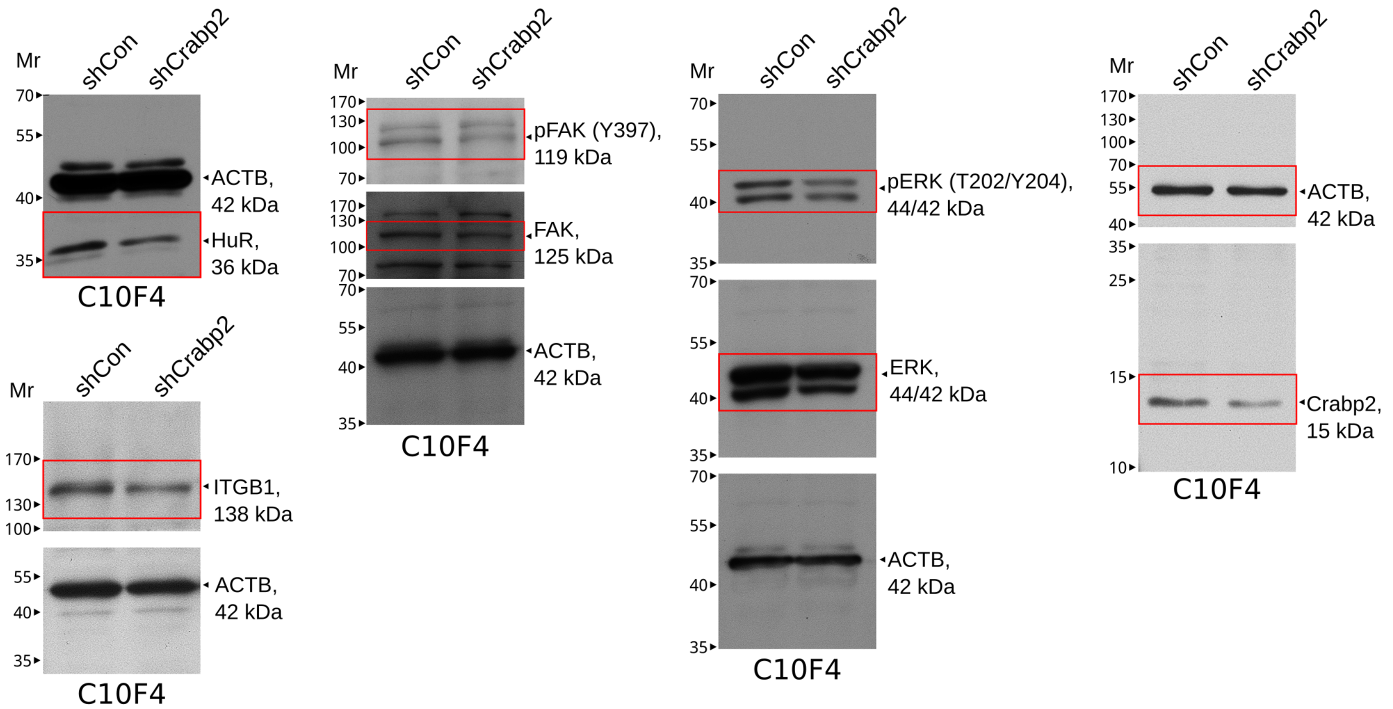

Mr
shCon
shCrabp2
70
55
40
35
ACTB, 42 kDa
HuR, 36 kDa
C10F4
Mr
shCon
shCrabp2
170
130
100
55
40
35
ITGB1, 138 kDa
ACTB, 42 kDa
C10F4
Mr
shCon
shCrabp2
170
130
100
70
pFAK (Y397), 119 kDa
FAK, 125 kDa
ACTB, 42 kDa
C10F4
Mr
shCon
shCrabp2
pERK (T202/Y204), 44/42 kDa
ERK, 44/42 kDa
ACTB, 42 kDa
C10F4
Mr
shCon
shCrabp2
25
15
10
ACTB, 42 kDa
Crabp2, 15 kDa
C10F4

Figure 4g

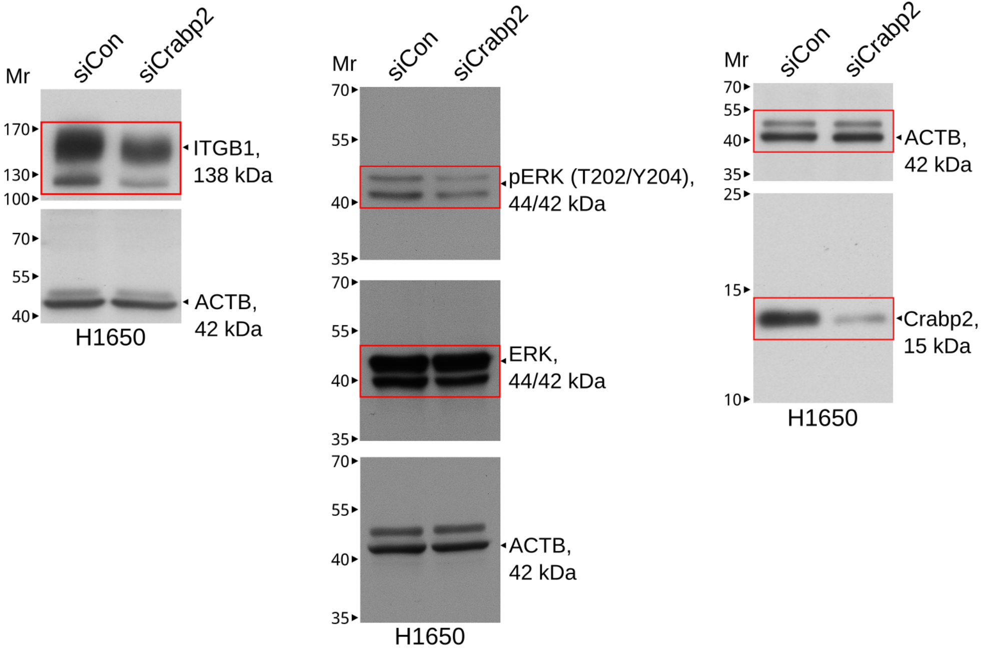

Mr
siCon
siCrabp2
170
130
100
ITGB1,
138 kDa
70
55
40
ACTB,
42 kDa
H1650
Mr
siCon
siCrabp2
70
55
40
35
pERK (T202/Y204),
44/42 kDa
70
55
40
35
ERK,
44/42 kDa
70
55
40
35
ACTB,
42 kDa
H1650
Mr
siCon
siCrabp2
70
55
40
35
ACTB,
42 kDa
25
15
10
Crabp2,
15 kDa
H1650

Figure 4h

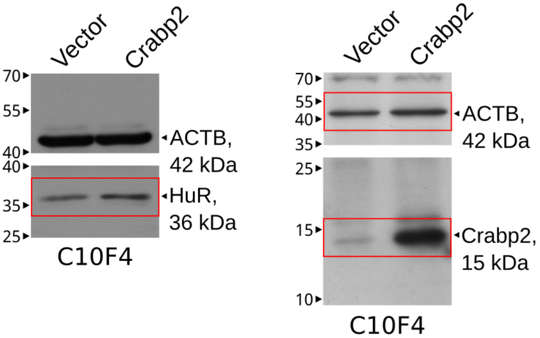

Vector
Crabp2
70
55
40
40
35
25
ACTB,
42 kDa
HuR,
36 kDa
C10F4
Vector
Crabp2
70
55
40
35
25
15
10
ACTB,
42 kDa
Crabp2,
15 kDa
C10F4

Figure 4i

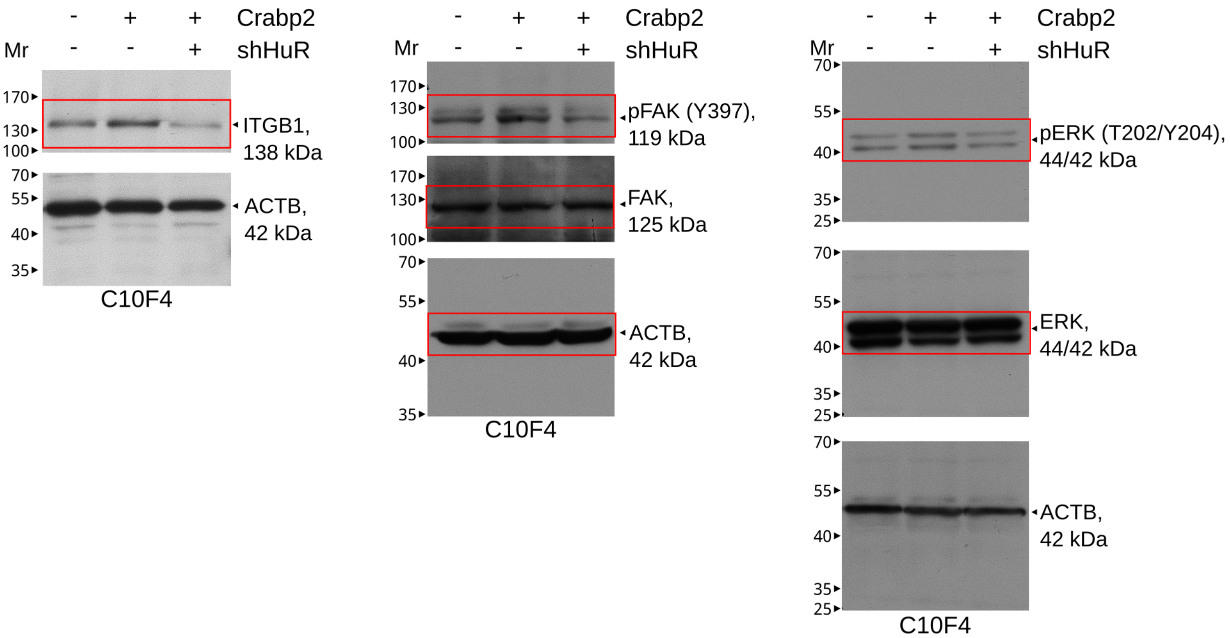

- + + Crabp2
Mr - - + shHuR
170
130
100
ITGB1,
138 kDa
70
55
40
35
ACTB,
42 kDa
C10F4
- + + Crabp2
Mr - - + shHuR
170
130
100
pFAK (Y397),
119 kDa
170
130
100
FAK,
125 kDa
70
55
40
35
ACTB,
42 kDa
C10F4
- + + Crabp2
Mr - - + shHuR
70
55
40
35
25
pERK (T202/Y204),
44/42 kDa
70
55
40
35
25
ERK,
44/42 kDa
70
55
40
35
25
ACTB,
42 kDa
C10F4

Figure 5a-b

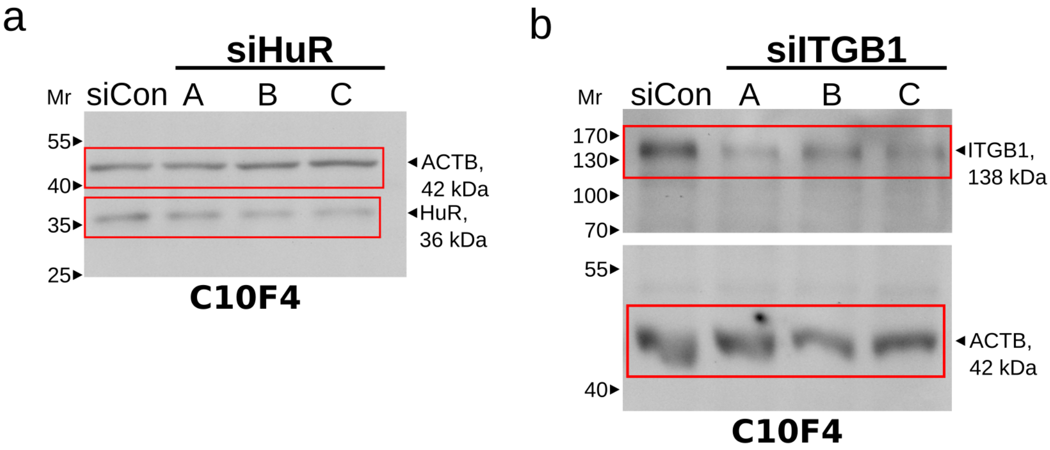

a
siHuR
Mr
siCon
A
B
C
55
40
35
25
ACTB,
42 kDa
HuR,
36 kDa
C10F4
b
siITGB1
Mr
siCon
A
B
C
170
130
100
70
ITGB1,
138 kDa
55
40
ACTB,
42 kDa
C10F4

Figure 7b

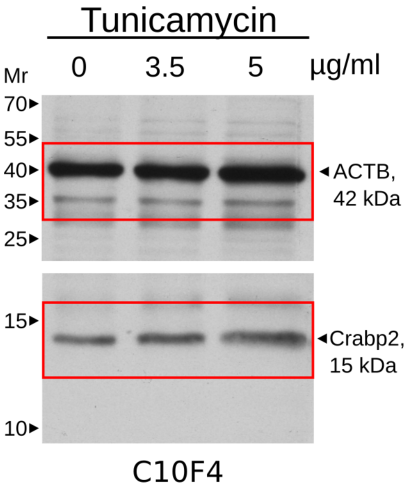
Tunicamycin
0
3.5
5
µg/ml
Mr
70
55
40
35
25
ACTB,
42 kDa
15
10
Crabp2,
15 kDa
C10F4

Figure S3

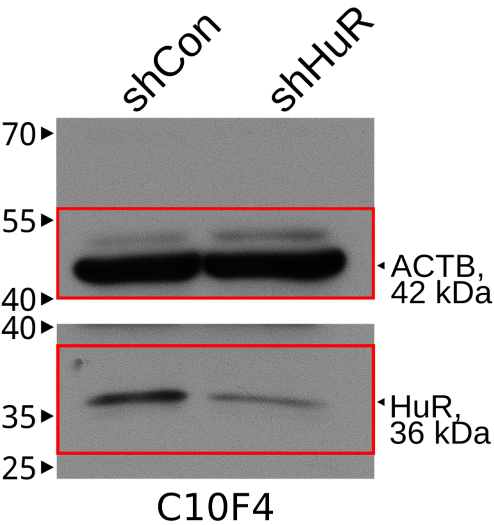

Supplement: Supplementary file 1 — Supplementary Information [file 41598_2018_37443_MOESM1_ESM.pdf]
